# Ring-Opening Copolymerization of Cyclohexene Oxide and Cyclic Anhydrides Catalyzed by Bimetallic Scorpionate Zinc Catalysts

**DOI:** 10.3390/polym13101651

**Published:** 2021-05-19

**Authors:** Felipe de la Cruz-Martínez, Marc Martínez de Sarasa Buchaca, Almudena del Campo-Balguerías, Juan Fernández-Baeza, Luis F. Sánchez-Barba, Andrés Garcés, Carlos Alonso-Moreno, José A. Castro-Osma, Agustín Lara-Sánchez

**Affiliations:** 1 Universidad de Castilla-La Mancha, Departamento de Química Inorgánica, Orgánica y Bioquímica-Centro de Innovación en Química Avanzada (ORFEO-CINQA), Facultad de Ciencias y Tecnologías Químicas, 13071 Ciudad Real, Spain; Felipe.Cruz@uclm.es (F.d.l.C.-M.); marc.martinezsarasa@uclm.es (M.M.d.S.B.); Juan.FBaeza@uclm.es (J.F.-B.); 2 Universidad de Castilla-La Mancha, Departamento de Química Inorgánica, Orgánica y Bioquímica-Centro de Innovación en Química Avanzada (ORFEO-CINQA), Facultad de Farmacia, 02071 Albacete, Spain; almudenadelcampo@gmail.com (A.d.C.-B.); Carlos.AMoreno@uclm.es (C.A.-M.); 3 Universidad Rey Juan Carlos, Departamento de Biología y Geología, Física y Química Inorgánica, 28933 Móstoles, Spain; luisfernando.sanchezbarba@urjc.es (L.F.S.-B.); andres.garces@urjc.es (A.G.)

**Keywords:** ring-opening copolymerization, epoxides, cyclic anhydrides, polymers, zinc complexes

## Abstract

The catalytic activity and high selectivity reported by bimetallic heteroscorpionate acetate zinc complexes in ring-opening copolymerization (ROCOP) reactions involving CO_2_ as substrate encouraged us to expand their use as catalysts for ROCOP of cyclohexene oxide (CHO) and cyclic anhydrides. Among the catalysts tested for the ROCOP of CHO and phthalic anhydride at different reaction conditions, the most active catalytic system was the combination of complex **3** with bis(triphenylphosphine)iminium as cocatalyst in toluene at 80 °C. Once the optimal catalytic system was determined, the scope in terms of other cyclic anhydrides was broadened. The catalytic system was capable of copolymerizing selectively and efficiently CHO with phthalic, maleic, succinic and naphthalic anhydrides to afford the corresponding polyester materials. The polyesters obtained were characterized by spectroscopic, spectrometric, and calorimetric techniques. Finally, the reaction mechanism of the catalytic system was proposed based on stoichiometric reactions.

## 1. Introduction

**Scheme 1:**
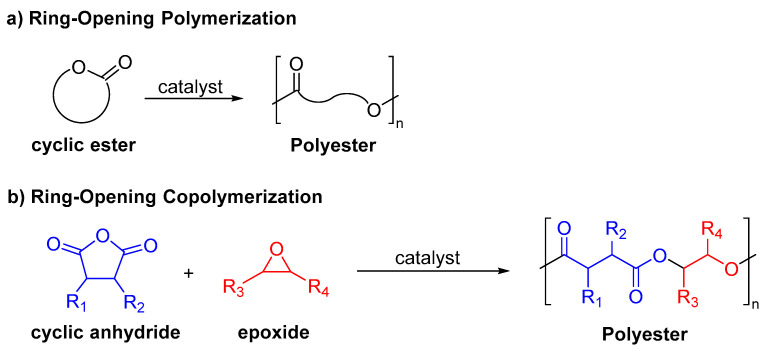
Synthesis of polyester materials via (**a**) ROP of cyclic esters; (**b**) ROCOP of epoxides and cyclic anhydrides.

Commercial polyester materials are currently produced by polycondensation reactions between diols and diacids at high reaction temperature and prolonged reaction times to afford high-molecular weight polyesters [1,2]. In recent years, a broad range of biodegradable polyesters have been obtained via ring-opening polymerization (ROP) of cyclic esters (Scheme 1a) as a more sustainable alternative to traditional polyester materials [3,4,5,6,7,8,9,10]. In fact, polycaprolactone and polylactide have already been used for several applications [11,12,13,14]. However, the microstructure and properties of the resulting polymers are restricted by the limited number of commercially available cyclic esters. In this regard, the ring-opening copolymerization (ROCOP) of epoxides and cyclic anhydrides (Scheme 1b) has emerged in recent years as alternative to the ROP of cyclic esters to prepare a larger number of polyester materials with a broader range of microstructures, properties and applications [15]. 

The ROCOP reaction of epoxides and cyclic anhydrides requires the use of a catalyst system, which is usually comprised by a combination of a metal complex and a nucleophile source [15]. A wide range of metal complexes, including chromium [16,17,18,19,20], magnesium [21,22,23], cobalt [24,25,26,27,28], manganese [26,29,30], iron [31,32,33,34], aluminum [3,35,36,37,38,39,40] and zinc [41,42,43,44,45,46,47] has been used as catalysts for this process. Most of these complexes required the use of a cocatalyst such as tetrabutylammonium salts (TBAX), bis-(triphenylphospine)iminium salts (PPNX) or 4-dimethylaminopyridine (DMAP) to increase the efficiency and selectivity of the metal complex [15]. In this context, zinc acetate complexes have displayed excellent catalytic activity and selectivity for the ROCOP reaction of epoxides and cyclic anhydrides. Coates et al. developed a very efficient β-diiminate zinc acetate complex for the ROCOP of different epoxides and bi- or tricyclic anhydrides to afford the corresponding polymers with high molecular weights and narrow polydispersities [48]. Williams et al. obtained several homo- and heterobimetallic zinc complexes with outstanding catalytic activity and selectivity towards the synthesis of well-controlled polyester materials [21,44,45]. These bimetallic catalysts can activate both monomers facilitating the nucleophilic attack of the alkoxide or carboxylate group of one zinc atom to the activated monomer on the second zinc center. Moreover, these complexes have also catalyzed the synthesis of a broad range of multiblock polymeric materials by ROCOP of epoxides, anhydrides, carbon dioxide or lactones [49,50]. Zinc amido-oxazolinate complexes [43,46] have also been tested as catalysts for the ROCOP of epoxides and cyclic anhydrides, obtaining TOF values up to 4000 h^−1^ [43]. It was observed that there is a relationship between catalytic activity and epoxide substrates used due to the effect of the electronic and steric properties of the epoxides in the chain propagation step [46]. Schiff-base zinc complexes in combination with a range of cocatalysts have been investigated as catalyst systems for the bulk and solution ROCOP of cyclohexene oxide (CHO) and maleic anhydride (MA) to produce polymeric materials [42]. The results showed alternating poly(cyclohexene maleate) when the process was carried out in toluene using a combination of zinc complex and DMAP as a cocatalyst. 

Scorpionate aluminum and zinc complexes have been developed as efficient catalysts for the ROCOP of carbon dioxide and epoxides to afford polycarbonates and the terpolymerization reaction of epoxides, cyclic anhydrides and carbon dioxide [3,36,51,52,53]. The zinc-based complexes displayed good catalytic activity and selectivity for the synthesis of medium molecular weight poly(cyclohexene carbonate) (PCHC) with narrow molecular weight distributions in the absence of a nucleophile as a cocatalyst [52,53]. The use of helical aluminum catalysts for the ROCOP of a range of epoxides and cyclic anhydrides or CHO and CO_2_ afforded the synthesis of polyester and polyether-polycarbonate materials respectively [36,51]. Furthermore, we have recently reported a new family of acetate zinc complexes as catalysts for the ROCOP of CHO with CO_2_ to afford polycarbonates or the terpolymerization of CHO, CO_2_ and phthalic anhydride (PA) to produce polyester-polycarbonate materials [53]. Encouraged by the catalytic activity and selectivity displayed by these complexes in ROCOP reactions involving CO_2_, in this work, we have studied the use of these friendly zinc complexes as catalysts for the ROCOP of CHO and cyclic anhydrides with the aim of extend the substrate scope for these complexes in the synthesis of polymeric materials. In addition, a mechanistic proposal has been presented for this catalytic process.

## 2. Materials and Methods

All manipulations were performed under nitrogen, using standard Schlenk techniques. Microanalyses were carried out with a Perkin–Elmer 2400 CHN analyzer (PerkinElmer, Inc., Waltham, MA, USA). ^1^H and ^13^C NMR spectra were recorded on a Bruker Ascend TM-500 spectrometer (Bruker, Billerica, MA, USA) and referenced to the residual deuterated solvent. Gel permeation chromatography (GPC) measurements were performed on a Shimadzu LC-20A instrument (Shimadzu Corporation, Kyoto, Japan) equipped with a TSK-GEL G3000H column and a refractive index detector (RID-20A). The GPC column was eluted with THF at 25 °C at 1 mL·min^−1^ and was calibrated using eight monodisperse polystyrene standards in the range 580–48,300 Da. TGA analysis was performed on a TA instrument TGA-Q50 (TA Instruments Inc., New Castle, DE, USA). The heating rate for the sample was 10 °C/min, and the nitrogen flow rate was 60 mL/min. DSC curves were obtained under N_2_ atmosphere on a TA Instrument DSC-Q20 (TA Instruments Inc., New Castle, DE, USA). Samples were weighed into aluminum crucibles with 5 mg of sample and subjected to two heating cycles at a heating rate of 10 °C/min. The MALDI-ToF spectra were acquired using a Bruker Autoflex II TOF/TOF spectrometer (Bruker, Billerica, MA, USA) using dithranol (1,8,9-trihydroxyanthracene) as matrix material and NaOAc as additive. Commercially available chemicals (Alfa Aesar, Ward Hill, MA, US, Sigma-Aldrich, Munich, Germany and Fluka, Buchs, Switzerland) were used as received. 

### 2.1. Materials and Reagents

Toluene was predried over sodium wire and distilled under nitrogen from sodium. Deuterated solvents were stored over activated 4 Å molecular sieves and degassed by several freeze-thaw cycles. The synthesis of ligand precursors and zinc complexes was carried out as previously reported [53,54,55]. Cyclohexene oxide (Sigma-Aldrich, Munich, Germany) was pre-dried over calcium hydride, distilled under vacuum and stored under nitrogen in a glove box. Phthalic, maleic and succinic anhydride (Sigma-Aldrich) were sublimed three times and naphthalic anhydride was recrystallized in chloroform and stored under nitrogen in a glove box. All other reagents were purchased from common commercial sources and used as received.

### 2.2. General Procedure for the ROCOP of CHO with Cyclic Anhydrides

In the glovebox, zinc complex (50.09 μmol), cocatalyst (0.01 mmol) and cyclic anhydride (5.09 mmol) were placed into a 10 mL Schlenk equipped with a small stir bar. Toluene (2 mL) and cyclohexene oxide (5.09 mmol) were added to the reaction mixture. Then, the Schlenk was taken out of the glovebox and placed in a preheated oil bath under a nitrogen atmosphere at the desired temperature. The conversion of cyclic anhydride into the corresponding polyester was monitored by NMR. Once the polyester was obtained, the viscous mixture was dissolved in the minimum amount of dichloromethane, precipitated with an excess of methanol to remove the unreacted reactants, catalyst, and co-catalyst and finally, was filtered off and dried to afford a white solid.

## 3. Results

**Figure 1:**
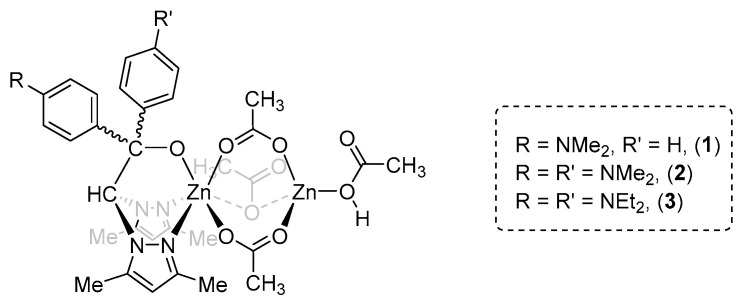
Chemical structure of complexes **1**−**3**.

Dinuclear zinc complexes **1**–**3** (Figure 1) were synthesized in excellent yields as previously reported [53]. These complexes showed high stability in solution and in solid-state in the presence of air for months. As previously commented, these complexes displayed good catalytic activity for the synthesis of polycarbonates from the reaction of cyclohexene oxide with carbon dioxide and for the preparation of terpolymers [53].

**Scheme 2:**
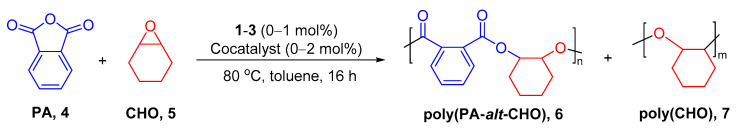
Synthesis of poly(PA-*alt*-CHO) (**6**) catalyzed by complexes **1**–**3**.

In order to extend the scope of application of these compounds in catalysis, further studies for ROCOP reactions were carried out. First, we explored the use of these complexes for the preparation of poly(PA-*alt*-CHO) (**6**) via ROCOP of phthalic anhydride (**4**) and cyclohexene oxide (**5**) (Scheme 2) and the results are shown in Table 1. Conversion and selectivity towards **6** were determined by ^1^H NMR spectroscopy of the crude reaction mixture.

Initially, the ROCOP reaction of **4** and **5** was investigated using 1 mol.% of complexes **1**–**3** at 80 °C for 16 h under solvent-free conditions (Table 1, entries 1–3). Under these reaction conditions, complexes **1**–**3** displayed good catalytic activity but low selectivity towards the synthesis of **6** probably due to the low solubility of the anhydride into the epoxide. Thus, in order to improve the solubility of PA into CHO, the concentration of CHO in the reaction mixture was increased. Unfortunately, 79–82% of polyether was obtained under these conditions (Table 1, entries 4–6). Importantly, bimetallic zinc complexes **1–3** required the addition of PA into the reaction mixture to catalyze the ROP of CHO (Table 1, entries 7–9).

The addition of toluene as a solvent resulted in an increase of the selectivity towards the formation of **6**, although the catalytic activity decreased (Table 1, entries 10–12). Complex **3** displayed the highest catalytic activity and therefore complex **3** was selected to optimize the catalytic system. Entries 12 and 13 of Table 1 show that a more coordinative solvent such as THF caused a decrease in the catalytic activity of the system. On the other hand, lower conversion was achieved when using nondistilled CHO (Table 1, entry 14). As it was expected, the addition of an external nucleophile such as of 4-dimethylaminopyridine (DMAP) not only increased the selectivity but also the catalytic activity of complex **3** (Table 1, entries 15 and 16) [3,38].

In order to continue with the optimization of the catalytic system for the synthesis of **6**, the addition of a range of nucleophiles was investigated using 1 mol.% of complex **3** and 2 mol.% of the corresponding cocatalyst in toluene at 80 °C for 16 h. The results are shown in Table 2. Tetrabutylammonium chloride (TBAC), bromide (TBAB) and iodide (TBAI) were initially screened as cocatalyst obtaining conversions in the range of 66 to 88% with excellent selectivities towards **6** (93–97%) (Table 2, entries 1–3). Then, bis(triphenylphosphine)iminium chloride (PPNCl), azide (PPNN_3_) and 2,4-dinitrophenolate (PPNDNP) were studied as cocatalysts (Table 2, entries 4–6) obtaining excellent conversions and selectivities towards **6**. In addition, when the reaction time was decreased to 8 h (Table 2, entries 8 and 9), higher conversion was obtained when using PPNCl as a cocatalyst (Table 2, entry 8). Finally, a control experiment using 2 mol.% of PPNCl in the absence of complex **3** (Table 2, entry 10) confirmed that both catalyst components are needed to achieve excellent conversion and selectivity. 

The effect of the catalyst loading on the catalytic activity, selectivity and molecular weight of the resulting copolymers was investigated (Table 3). When the catalyst loading was increased to 2 mol.% (Table 3, entry 1), quantitative conversion and 97% selectivity towards **6** were achieved. On the other hand, lower conversions and good selectivities were obtained when the catalyst loading was decreased (Table 3, entries 3–4). As can be seen in Table 3, low molecular weights (*M*_n_ = 2.46–3.67 kg/mol) and narrow polydispersity values (1.39–1.58) were obtained. Moreover, the reduction of the catalyst loading resulted in a slight increase in the molecular weight of the polyesters obtained (Table 3, entries 1–4).

**Scheme 3:**
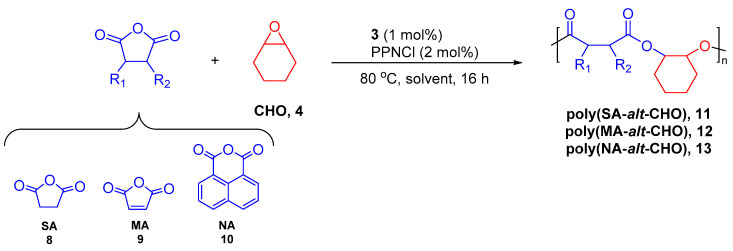
ROCOP of CHO and different cyclic anhydrides catalyzed by complex **3** and PPNCl.

Having determined the optimal reaction conditions, the use of several cyclic anhydrides was examined for the ROCOP process using CHO as epoxide substrate (Scheme 3) and the results are listed in Table 4. When succinic or maleic anhydride were used as the cyclic anhydride substrate, the ROCOP proceeded smoothly and excellent conversions and selectivities were obtained, affording the corresponding low to medium molecular weight polyester materials with narrow polydispersities (Table 4, entries 1–4). However, the use of naphthalic anhydride as monomer for the ROCOP with CHO was challenging (Table 4, entries 5–7). When the reaction was carried out in toluene at 80 °C, only 55% conversion of NA into poly(NA-*alt*-CHO) (**13**) was obtained (Table 4, entry 5). Therefore, the ROCOP of CHO and NA was studied using CHO as solvent. As can be seen in Table 4 (entries 6 and 7), higher conversions were achieved under these reaction conditions. Furthermore, all polyesters displayed low to medium molecular weights which were influenced by the solvent used in the process. 

**Figure 2:**
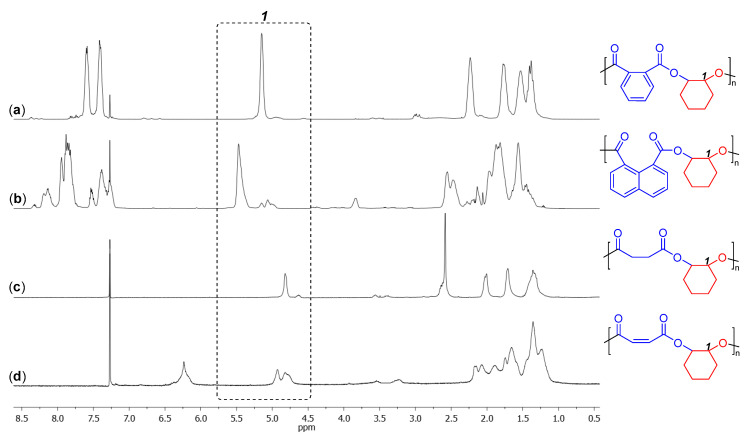
^1^H NMR spectra of (**a**) poly(PA-*alt*-CHO), Table 3, entry 2. (**b**) poly(NA-*alt*-CHO), Table 4, entry 6. (**c**) poly(SA-*alt*-CHO), Table 4, entry 1. (**d**) poly(MA-*alt*-CHO), Table 4, entry 3.

**Figure 3:**
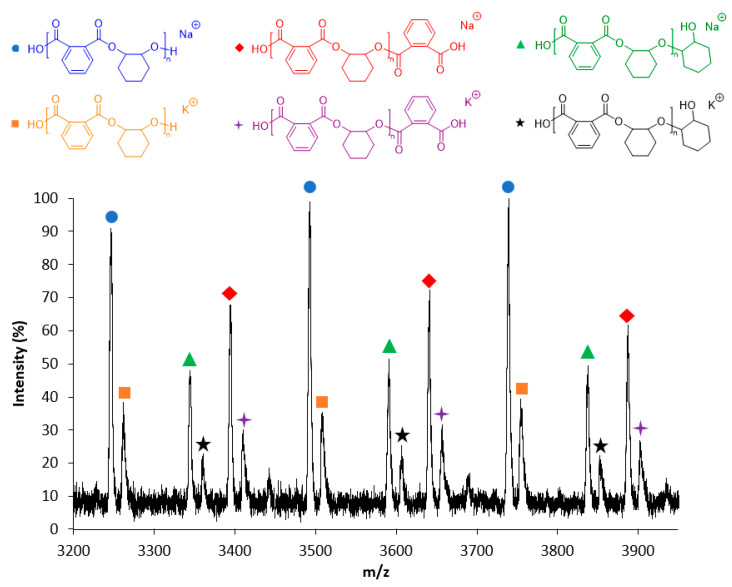
MALDI-ToF-MS spectrum of poly(PA-*alt*-CHO) **6** obtained using zinc complex **3** and PPNCl as catalyst system (Table 4, entry 2).

All polymers were characterized by NMR and IR spectroscopy (Figure 2 and Figures S1–S12 in the SI), gel permeation chromatography (GPC) (Figure S13 in the SI) and MALDI-ToF mass spectroscopy (Figure 3 and Figure S14 in the SI). The NMR spectra of copolymers **6**,**11**–**13** are in good agreement with those previously reported [32,36] and confirmed the formation of the corresponding alternating copolyester (Figure 2). On the other hand, the IR spectra of copolymers clearly showed the presence of a strong band in the range 1710–1729 cm^−1^ corresponding to the ν(C=O) stretching vibration of the ester group, which is shifted to lower wavenumbers compared with that of the corresponding cyclic anhydride starting materials (1736–1777 cm^−1^). 

MALDI-ToF analysis supported the formation of alternating poly(PA-*alt*-CHO) without the presence of polyether chains (Figure 3). The MALDI-ToF spectrum showed three major distributions of poly(PA-*alt*-CHO) end-capped with a carboxylic acid end-group and a hydroxyl, a cyclohexanol or a carboxylic acid end-groups with sodium or potassium as counterion. Even though the chloride initiates the copolymerization, no chloride end-group was shown due to the resulting acyl chloride was unstable and quickly hydrolyzed to the corresponding carboxylic acid during the work-up procedure.

Inductively coupled plasma–mass spectrometry (ICP–MS) was used to determine the metal content of the polyester synthesized in Table 3, entry 2. The Zn content of the polymer was determined to be 1.700 mg·g^−1^ by ICP–MS, confirming that most of the catalyst was removed in the purification process. However, this result showed that it is impossible to entirely remove the metal traces from the polymer when using a homogeneous catalyst.

**Figure 4:**
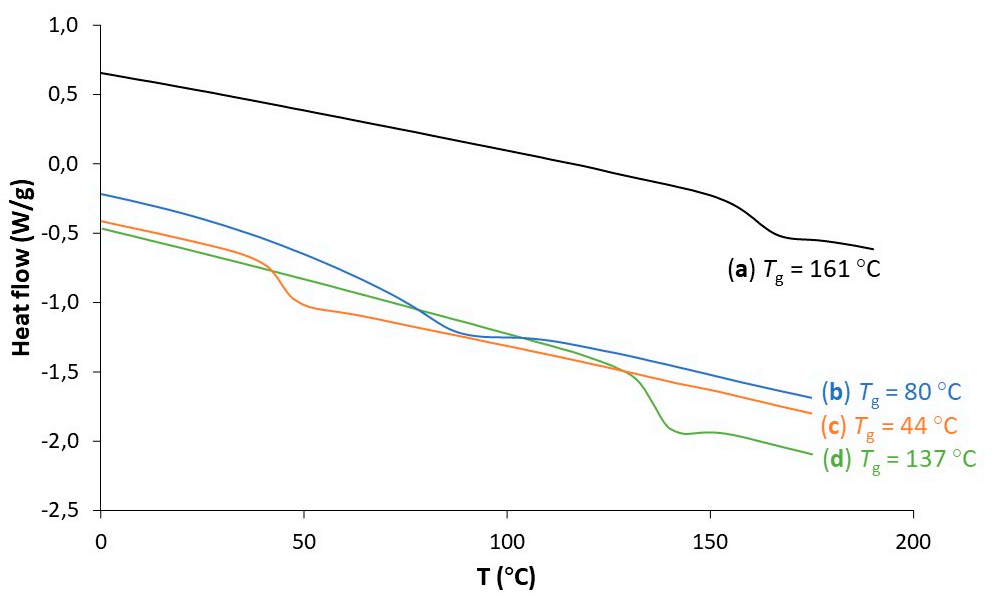
Selected regions of the DSC traces of (**a**) poly(NA-*alt*-CHO), Table 4, entry 6. (**b**) poly(MA-*alt*-CHO), Table 3, entry 2. (**c**) poly(SA-*alt*-CHO), Table 4, entry 3. (**d**) poly(PA-*alt*-CHO), Table 4, entry 1.

The thermal properties of the resulting polyesters were investigated by differential scanning calorimetry (DSC) (Figures S16, S18, S20 and S22 in the SI). As can be seen in Figure 4, the glass transition temperature (*T*_g_) of the copolymers is highly dependent on the substrate used. Thus, poly(SA-*alt*-CHO) and poly(MA-*alt*-CHO) displayed lower *T*_g_ values than the polyesters in which an aromatic anhydride was used. Moreover, poly(NA-*alt*-CHO) showed the highest *T*_g_ value because of the stiffness of the polyester. TGA analysis of polyesters **6**, **11**–**13** showed that all polyesters decompose around 300 °C (Figures S15, S17, S19 and S21 in the SI).

**Figure 5:**
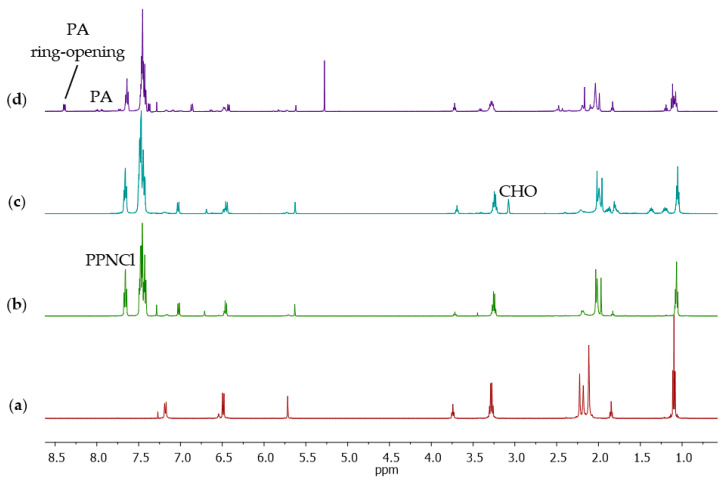
(**a**) ^1^H NMR spectrum of compound **3** in CDCl_3_. (**b**) ^1^H NMR spectrum of compound **3** + PPNCl in CDCl_3_. (**c**) ^1^H NMR spectrum of compound **3** + PPNCl + cyclohexene oxide **4** at 80 °C and t = 24 h in CDCl_3_. (**d**) ^1^H NMR spectrum of compound **3** + PPNCl + phthalic anhydride **5** at 80 °C and t = 24 h in CDCl_3_.

Then, several stoichiometric reactions were carried out in order to gain insight into the reaction mechanism for the ROCOP of CHO and PA catalyzed by the catalytic system comprised by complex **3** and PPNCl (Figure 5). Addition of two equivalents of PPNCl to a solution of complex **3** in CDCl_3_ resulted in minor changes in the NMR spectrum of complex **3** (Figure 5b). When **4** was added to the catalytic system, no changes in the NMR spectrum were observed after 24 h of reaction at 80 °C (Figure 5c). On the contrary, new signals showed up at around 8.5 ppm when PA was added to the catalytic system, suggesting that the first step of the copolymerization corresponds with the ring-opening of the cyclic anhydride (Figure 5d). 

**Figure 6:**
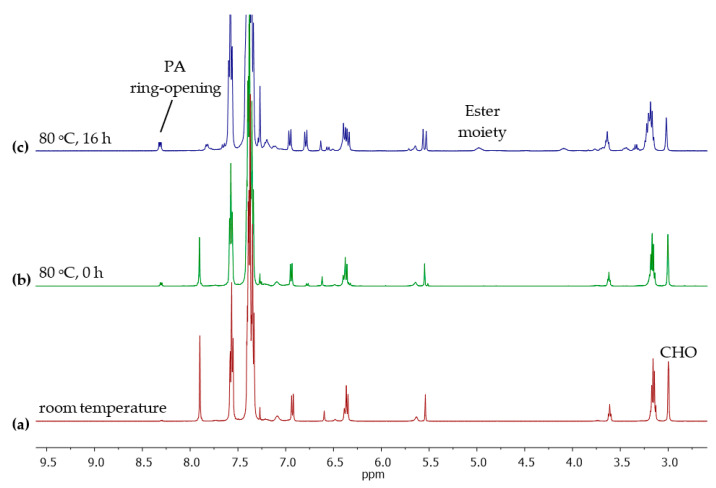
Reaction one equivalent of cyclohexene oxide **4** and phthalic anhydride **5** with one equivalent of complex **3** and two equivalents of PPNCl in CDCl_3_, (**a**) at room temperature; (**b**) at 80 °C after 0 h; (**c**) at 80 °C after 16 h.

The reaction of one equivalent of **4** and **5** with one equivalent of complex **3** and 2 equivalents of PPNCl at 80 °C was also monitored by ^1^H NMR (Figure 6). It is worth noting that even at room temperature phthalic anhydride was activated by the catalytic system, and the chloride anion provided by PPNCl was able to ring-open the activated anhydride as it is supported by the appearance of new resonance signals at 8.3 ppm. When the reaction temperature was increased up to 80 °C, the signal at 8.30 ppm increased its intensity, ascertaining the fact that the first step of the copolymerization is the ring-opening of the cyclic anhydride. On the other hand, no changes in the signal of CHO were observed within 16 h. After that, most of the cyclic anhydride was ring-opened and a new signal at 4.98 ppm showed up which corresponds to the ester moiety (Figure 6).

**Scheme 4:**
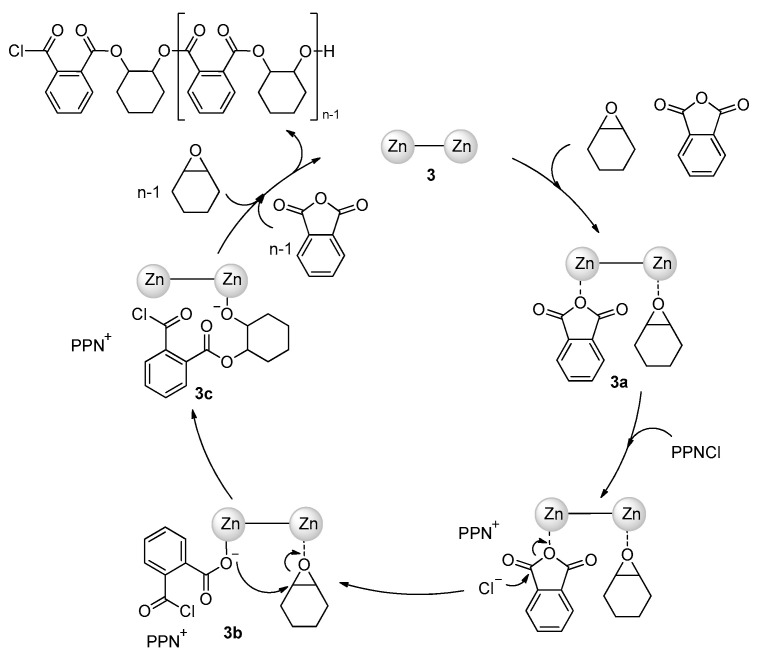
Proposed mechanism for the ROCOP of CHO and PA catalyzed by complex **3** and PPNCl.

A plausible mechanism for the ROCOP of CHO and PA catalyzed by the catalytic system comprised by complex **3** and PPNCl is proposed in Scheme 4. Firstly, the epoxide and cyclic anhydride may coordinate to the two zinc centers to form intermediate **3a**. Then, the chloride anion could attack at the activated cyclic anhydride to generate the metal carboxylate specie **3b** which can therefore ring-open the activated molecule of CHO by the second zinc center to give rise to the alkoxide specie **3c**, which could in turn experiment consecutive ring-opening reactions of cyclic anhydrides and epoxides to afford the corresponding polyester. Even though this proposal could be the main mechanism, an initiation step by an acetate group to result in polymers with an acetate end-group cannot be ruled out.

## 4. Conclusions

Bimetallic acetate zinc catalysts **1**–**3** were investigated as catalysts for the ROCOP of cyclohexene oxide and phthalic anhydride to obtain the corresponding poly(PA-*alt*-CHO). To optimize the copolymerization process, the effect of the solvent, temperature, type of cocatalyst, catalyst/cocatalyst ratio, and catalyst loading were studied. The combination of 1 mol.% of complex **3** and 2 mol.% of PPNCl at 80 °C for 16 h using toluene as solvent resulted to be an efficient catalyst system for ROCOP of CHO and PA with conversion and selectivity values up to 100% and 97%, respectively. This catalyst system also catalyzed ROCOP of CHO with succinic, maleic and naphthalic anhydrides to give rise to several polyester materials with low to medium molecular weight and narrow polydispersities. Stoichiometric reactions suggested that the first step of the copolymerization process is the ring-opening of the cyclic anhydride by the chloride counterion. Further catalyst development is ongoing in our research group to increase the catalytic activity and productivity of bimetallic zinc complexes as catalysts for ring-opening copolymerization processes. 

## Figures and Tables

**Table 1:** Synthesis of poly(PA-*alt*-CHO) **6** catalyzed by complexes **1**–**3** ^1^.

Entry	[Cat]:[CHO]:[PA]	Catalyst	Solvent	Conversion (%) ^2^	Polyester, 6 (%) ^2^	Polyether, 7 (%) ^2^
1	1:100:100	**1**	-	70	22	78
2	1:100:100	**2**	-	75	20	80
3	1:100:100	**3**	-	76	28	72
4	1:200:100	**1**	-	75	20	80
5	1:200:100	**2**	-	77	18	82
6	1:200:100	**3**	-	78	21	79
7	1:100:0	**1**	-	0	-	-
8	1:100:0	**2**	-	0	-	-
9	1:100:0	**3**	-	0	-	-
10	1:100:100	**1**	Toluene	42	42	58
11	1:100:100	**2**	Toluene	42	43	58
12	1:100:100	**3**	Toluene	50	43	57
13	1:100:100	**3**	THF	35	46	54
14 ^3^	1:100:100	**3**	Toluene	35	45	55
15 ^4^	1:100:100	**3**	Toluene	57	90	10
16 ^5^	1:100:100	**3**	Toluene	90	95	5

^1^ Reactions were carried out at 80 °C for 16 h using 1 mol.% of complexes **1**–**3** as catalysts. ^2^ Conversion relative to PA was determined by ^1^H NMR spectroscopy. ^3^ Nondistilled CHO was used. ^4^ 1 mol.% of 4-dimethylaminopyridine was used as cocatalyst. ^5^ 2 mol.% of 4-dimethylaminopyridine was used as cocatalyst.

**Table 2:** Influence of the cocatalyst in the optimization of the catalytic system for the synthesis of poly(PA-*alt*-CHO) (**6**) ^1^.

Entry	[3]:[Cocat]:[CHO]:[PA]	Cocatalyst	Conversion (%) ^2^	Polyester, 6 (%) ^2^	Polyether, 7 (%) ^2^
1	1:2:100:100	TBAC	84	97	3
2	1:2:100:100	TBAB	88	93	7
3	1:2:100:100	TBAI	66	97	3
4	1:2:100:100	PPNCl	96	97	3
5	1:2:100:100	PPNN_3_	66	95	5
6	1:2:100:100	PPNDNP	95	96	4
7	1:2:100:100	DMAP	90	95	5
8 ^3^	1:2:100:100	PPNCl	60	94	6
9 ^3^	1:2:100:100	PPNDNP	45	93	7
10 ^4^	0:2:100:100	PPNCl	40	96	4

^1^ Reactions were carried out in toluene (2 mL) at 80 °C for 16 h using complex **3** and a cocatalyst. ^2^ Conversion relative to PA was determined by ^1^H NMR spectroscopy. ^3^ Reaction was carried out for 8 h. ^4^ Complex **3** was not added.

**Table 3:** Effect of the catalyst loading on the activity of the catalytic system for the synthesis of poly(PA-*alt*-CHO) (**6**) ^1^.

Entry	[3]:[PPNCl]:[CHO]:[PA]	Conversion (%) ^2^	Polyester, 6 (%) ^2^	M_n exp_ (kg·mol^−1^) (PDI) ^3^
1	1:2:50:50	100	97	2.46 (1.58)
2	1:2:100:100	96	97	2.87 (1.49)
3	1:2:150:150	76	95	3.29 (1.45)
4	1:2:200:200	70	91	3.67 (1.39)

^1^ Reactions were carried out in toluene (2 mL) at 80 °C for 16 h using complex **3** and PPNCl. ^2^ Conversion relative to PA determined by ^1^H NMR spectroscopy. ^3^ Determined by GPC.

**Table 4:** Synthesis of polyesters **11–13** catalyzed by complex **3** and PPNCl ^1^.

Entry	[3]:[PPNCl]:[CHO]:[CA]	CA	Conversion (%) ^2^	Polyester, 6 (%) ^2^	*M*_n exp_ (kg·mol^−1^)(PDI) ^3^
1	1:2:100:100	SA	100	97	2.95 (1.43)
2	1:2:200:200	SA	99	99	4.03 (1.34)
3	1:2:100:100	MA	100	89	2.40 (1.51)
4	1:2:200:200	MA	100	90	3.52 (1.39)
5	1:2:100:100	NA	55	96	2.07 (1.48)
6 ^4^	1:2:100:100	NA	100	96	2.92 (1.45)
7 ^4^	1:2:200:200	NA	98	98	3.89 (1.37)

^1^ Reactions were carried out in toluene (2 mL) at 80 °C for 16 h using complex **3** and PPNCl. ^2^ Conversion relative to the cyclic anhydride determined by ^1^H NMR spectroscopy. ^3^ Determined by GPC. ^4^ Reactions were carried out using CHO (2 mL) as solvent.

## Supplementary Materials

The following are available online at https://www.mdpi.com/article/10.3390/polym13101651/s1, Figure S1. ^1^H NMR spectrum of poly(PA-*alt*-CHO) (**6**), Figure S2. ^13^C{^1^H} NMR spectrum of poly(PA-*alt*-CHO) (**6**), Figure S3. IR spectra of: poly(PA-*alt*-CHO) (**6**), PA (**4**), Figure S4. ^1^H NMR spectrum of poly(SA-*alt*-CHO) (**11**), Figure S5. ^13^C{^1^H} NMR spectrum of poly(SA-*alt*-CHO) (**11**), Figure S6. IR spectra of: (a) poly(SA-*alt*-CHO) (**11**), (b) SA (**8**), Figure S7. ^1^H NMR spectrum of poly(MA-*alt*-CHO) (**12**), Figure S8. ^13^C{^1^H} NMR spectrum of poly(MA-*alt*-CHO) (**12**), Figure S8. ^13^C{^1^H} NMR spectrum of poly(MA-*alt*-CHO) (**12**), Figure S9. IR spectra of: (a) poly(MA-*alt*-CHO) (**12**), (b) MA (**9**), Figure S10. ^1^H NMR spectrum of poly(NA-*alt*-CHO) (**13**), Figure S11. ^13^C{^1^H} NMR spectrum of poly(NA-*alt*-CHO) (**13**), Figure S12. IR spectra of: (a) poly(NA-*alt*-CHO) (**13**), (b) NA (**10**), Figure S13. GPC profile of poly(PA-*alt*-CHO) (**6**), Figure S14. MALDI-ToF spectrum of poly(PA-*alt*-CHO) (**6**), Figure S15. TGA analysis of poly(PA-*alt*-CHO) (**6**), Figure S16. DSC analysis of poly(PA-*alt*-CHO) (**6**), Figure S17. TGA analysis of poly(SA-*alt*-CHO) (**11**), Figure S18. DSC analysis of poly(SA-*alt*-CHO) (**11**), Figure S19. TGA analysis of poly(MA-*alt*-CHO) (**12**), Figure S20. DSC analysis of poly(MA-*alt*-CHO) (**12**), Figure S21. TGA analysis of poly(NA-*alt*-CHO) (**13**), Figure S22. DSC analysis of poly(NA-*alt*-CHO) (**13**).
