# Supplementary material for: Ring-Opening Copolymerization of Cyclohexene Oxide and Cyclic Anhydrides Catalyzed by Bimetallic Scorpionate Zinc Catalysts
Source: Polymers (Basel). 2021 May 19;13(10):1651. doi: 10.3390/polym13101651 (PMC8161297; doi:10.3390/polym13101651)

SUPPORTING INFORMATION FOR:

# Ring-Opening Copolymerization of Cyclohexene Oxide and Cyclic Anhydrides catalyzed by Bimetallic Scorpionate Zinc Catalysts

*Felipe de la Cruz-Martínez [1], Marc Martínez de Sarasa Buchaca [1], Almudena del Campo-Balguerías[2], Juan Fernández-Baeza [1], Luis F. Sánchez-Barba,[3] Andrés Garcés[3], Carlos Alonso-Moreno [2], José A. Castro-Osma [2,*] and Agustín Lara-Sánchez [1,*]*

[1] Universidad de Castilla-La Mancha, Departamento de Química Inorgánica, Orgánica y Bioquímica-Centro de Innovación en Química Avanzada (ORFEO-CINQA), Facultad de Ciencias y Tecnologías Químicas, 13071-Ciudad Real, Spain; e-mail: Agustin.Lara@uclm.es

[2] Universidad de Castilla-La Mancha, Departamento de Química Inorgánica, Orgánica y Bioquímica-Centro de Innovación en Química Avanzada (ORFEO-CINQA), Facultad de Farmacia, 02071-Albacete, Spain. e-mail: JoseAntonio.Castro@uclm.es.

[3] Universidad Rey Juan Carlos, Departamento de Biología y Geología, Física y Química Inorgánica, 28933-Móstoles, Spain.

* Correspondence: Agustin.Lara@uclm.es, JoseAntonio.Castro@uclm.es

## Table of Contents

**Figure S1**. $^{1}$H NMR spectrum of poly(PA-*alt*-CHO) (**6**) S3

**Figure S2**. $^{13}$C{$^{1}$H} NMR spectrum of poly(PA-*alt*-CHO) (**6**) S3

**Figure S3**. IR spectra of: a) poly(PA-*alt*-CHO) (**6**), b) PA (**4**) S4

**Figure S4.** $^{1}$H NMR spectrum of poly(SA-*alt*-CHO) (**11**) S5

**Figure S5**. $^{13}$C{$^{1}$H} NMR spectrum of poly(SA-*alt*-CHO) (**11**) S5

**Figure S6**. IR spectra of: a) poly(SA-*alt*-CHO) (**11**), b) SA (**8**) S6

**Figure S7**. $^{1}$H NMR spectrum of poly(MA-*alt*-CHO) (**12**) S7

**Figure S8**. $^{13}$C{$^{1}$H} NMR spectrum of poly(MA-*alt*-CHO) (**12**) S7

**Figure S9**. IR spectra of: a) poly(MA-*alt*-CHO) (**12**), b) MA (**9**) S8

**Figure S10**. $^{1}$H NMR spectrum of poly(NA-*alt*-CHO) (**13**) S9

**Figure S11**. $^{13}$C{$^{1}$H} NMR spectrum of poly(NA-*alt*-CHO) (**13**) S9

**Figure S12**. IR spectra of: a) poly(NA-*alt*-CHO) (**13**), b) NA (**10**) S10

**Figure S13**. GPC profile of poly(PA-*alt*-CHO) (**6**) S11

**Figure S14**. MALDI-ToF spectrum of poly(PA-*alt*-CHO) (**6**) S12

**Figure S15**. TGA analysis of poly(PA-*alt*-CHO) (**6**) S13

**Figure S16**. DSC analysis of poly(PA-*alt*-CHO) (**6**) S13

**Figure S17**. TGA analysis of poly(SA-*alt*-CHO) (**11**) S14

**Figure S18**. DSC analysis of poly(SA-*alt*-CHO) (**11**) S14

**Figure S19**. TGA analysis of poly(MA-*alt*-CHO) (**12**) S15

**Figure S20**. DSC analysis of poly(MA-*alt*-CHO) (**12**) S15

**Figure S21**. TGA analysis of poly(NA-*alt*-CHO) (**13**) S16

**Figure S22**. DSC analysis of poly(NA-*alt*-CHO) (**13**) S16

**Figure S1**. $^{1}$H NMR spectrum of poly(PA-*alt*-CHO) (**6**)

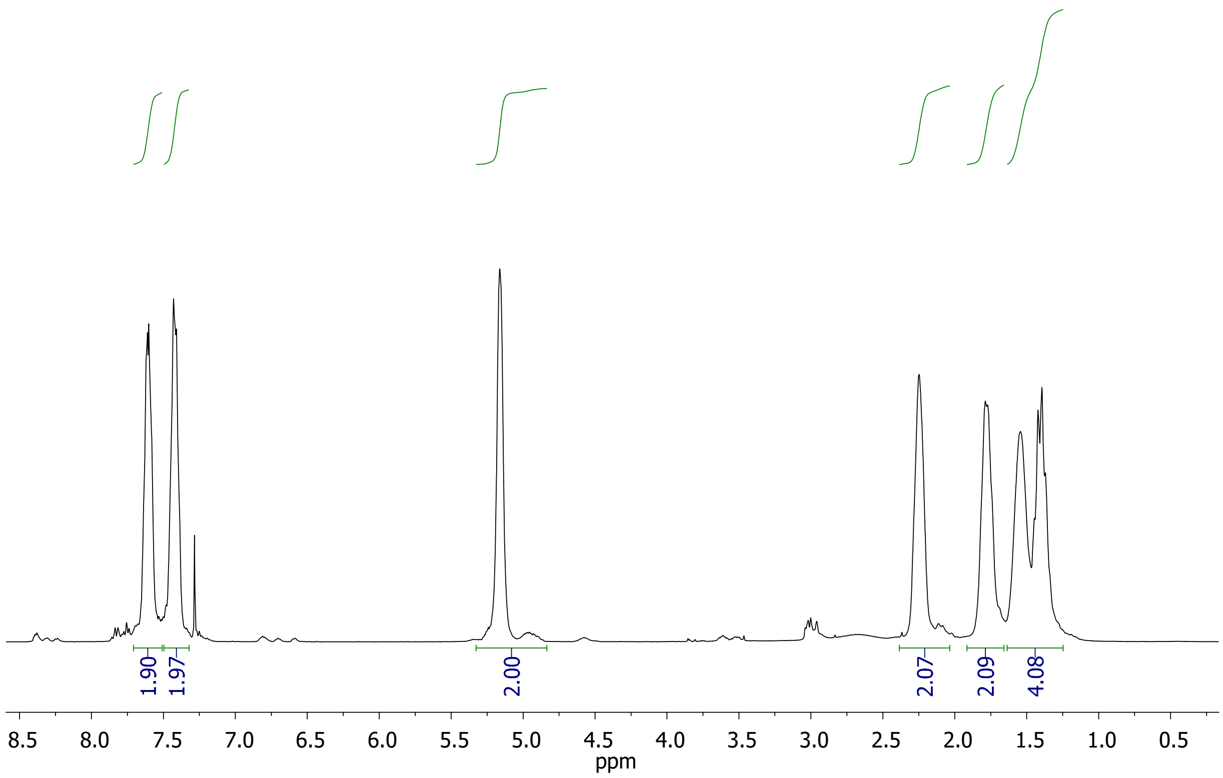


**Figure S2**. $^{13}$C{$^{1}$H} NMR spectrum of poly(PA-*alt*-CHO) (**6**)

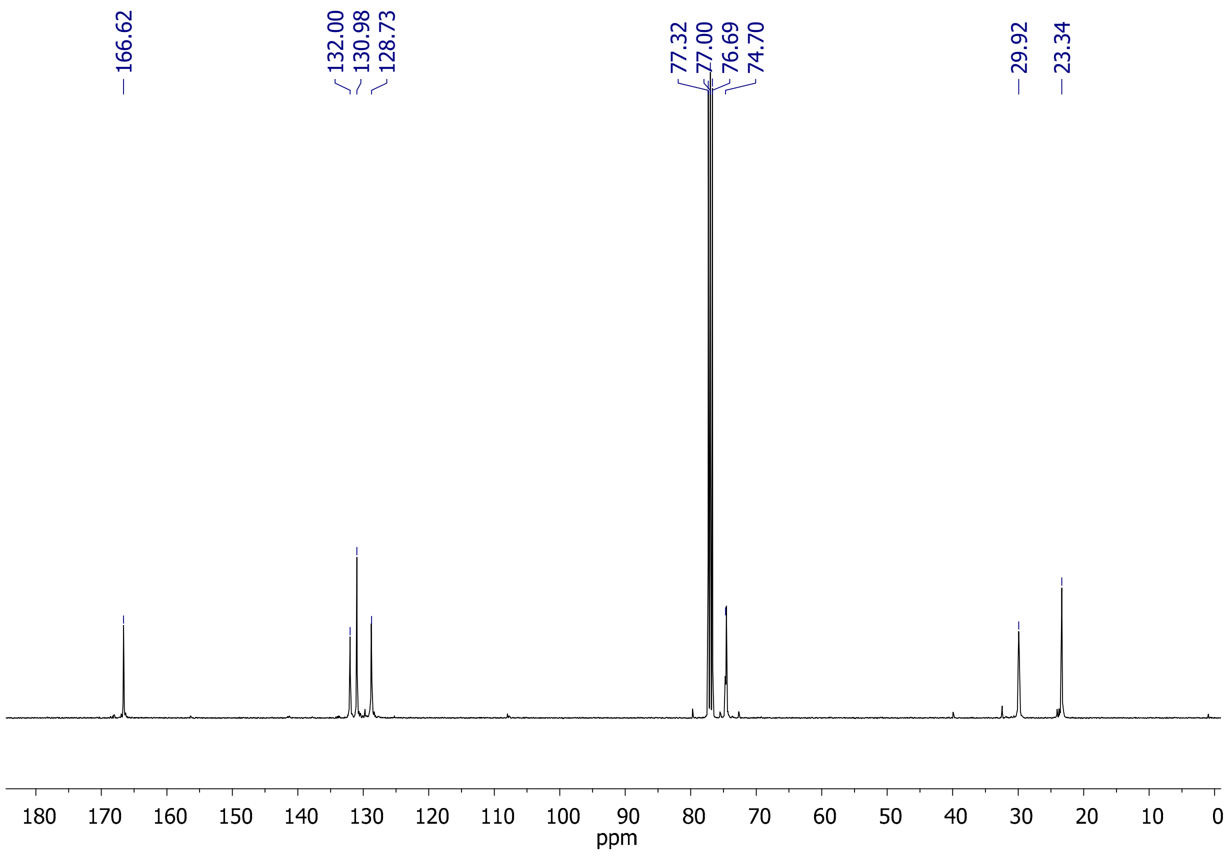

**Figure S3**. IR spectra of: a) poly(PA-*alt*-CHO) (**6**), b) PA (**4**)

**a)**

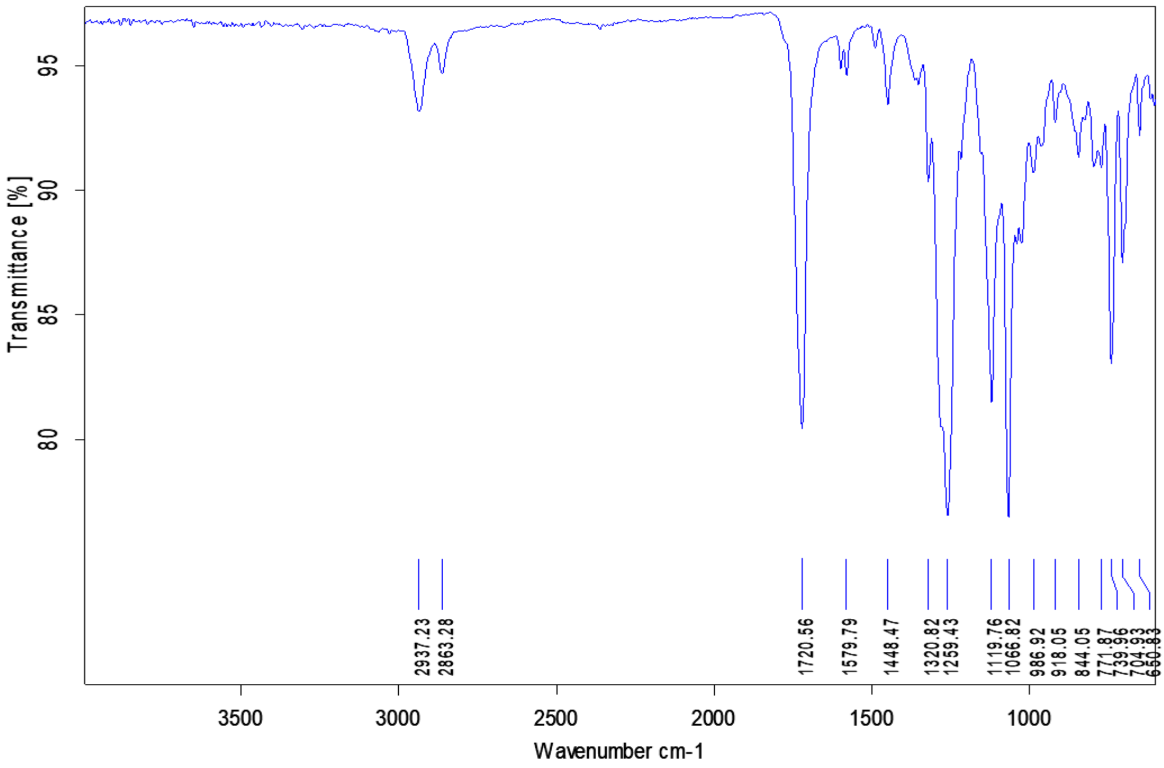


**b)**

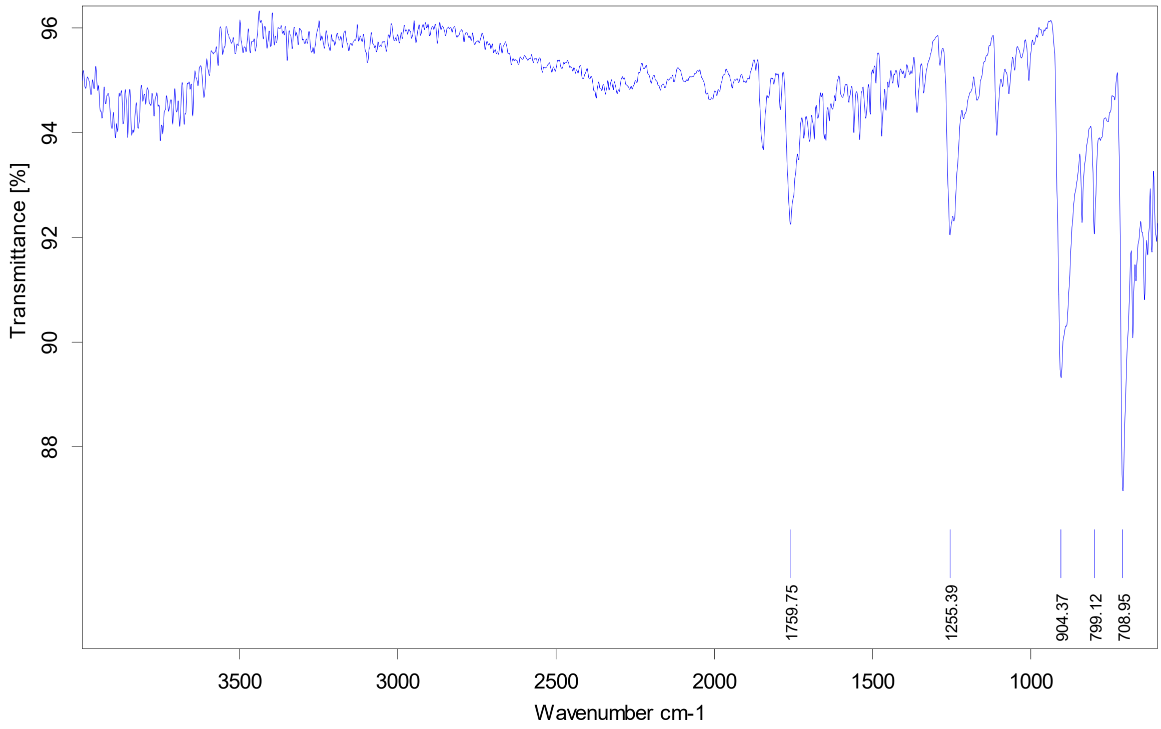

**Figure S4.** $^{1}$H NMR spectrum of poly(SA-*alt*-CHO) (**11**)

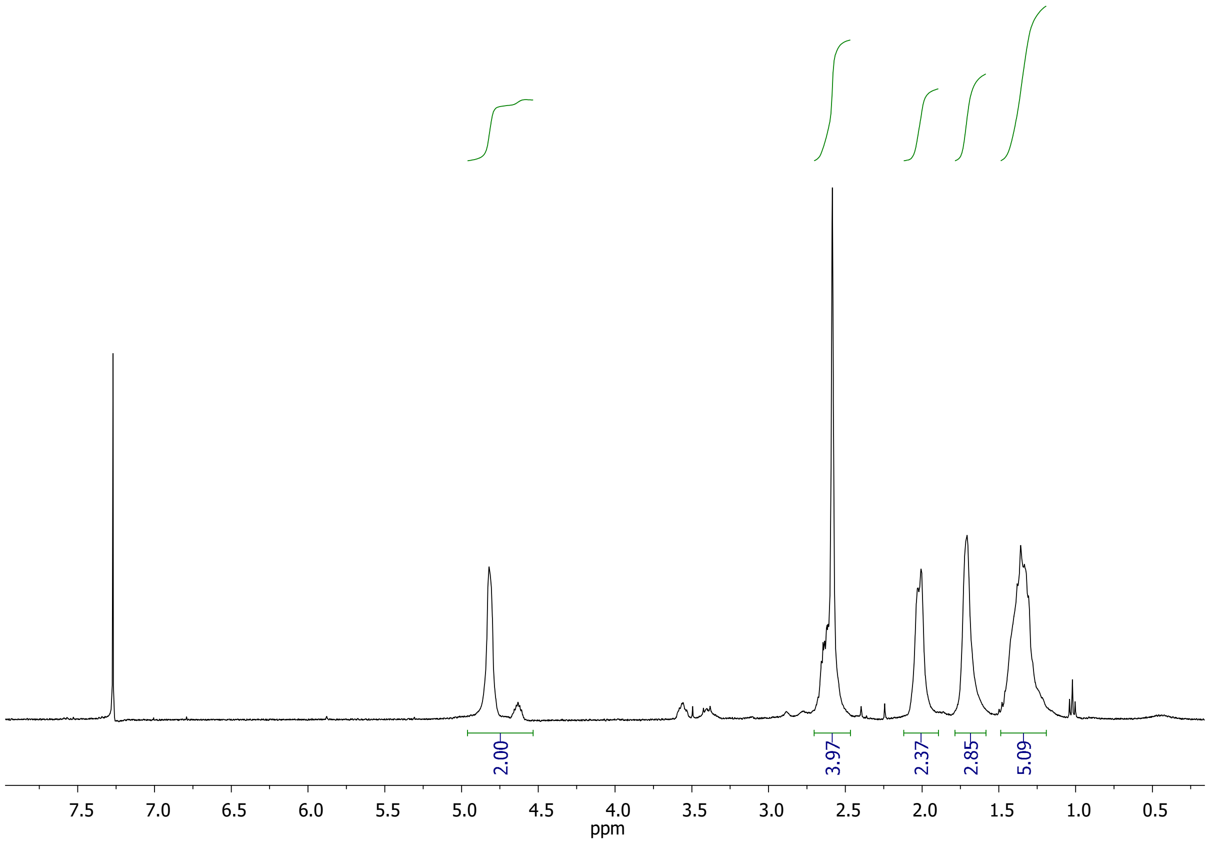


**Figure S5**. $^{13}$C{$^{1}$H} NMR spectrum of poly(SA-*alt*-CHO) (**11**)

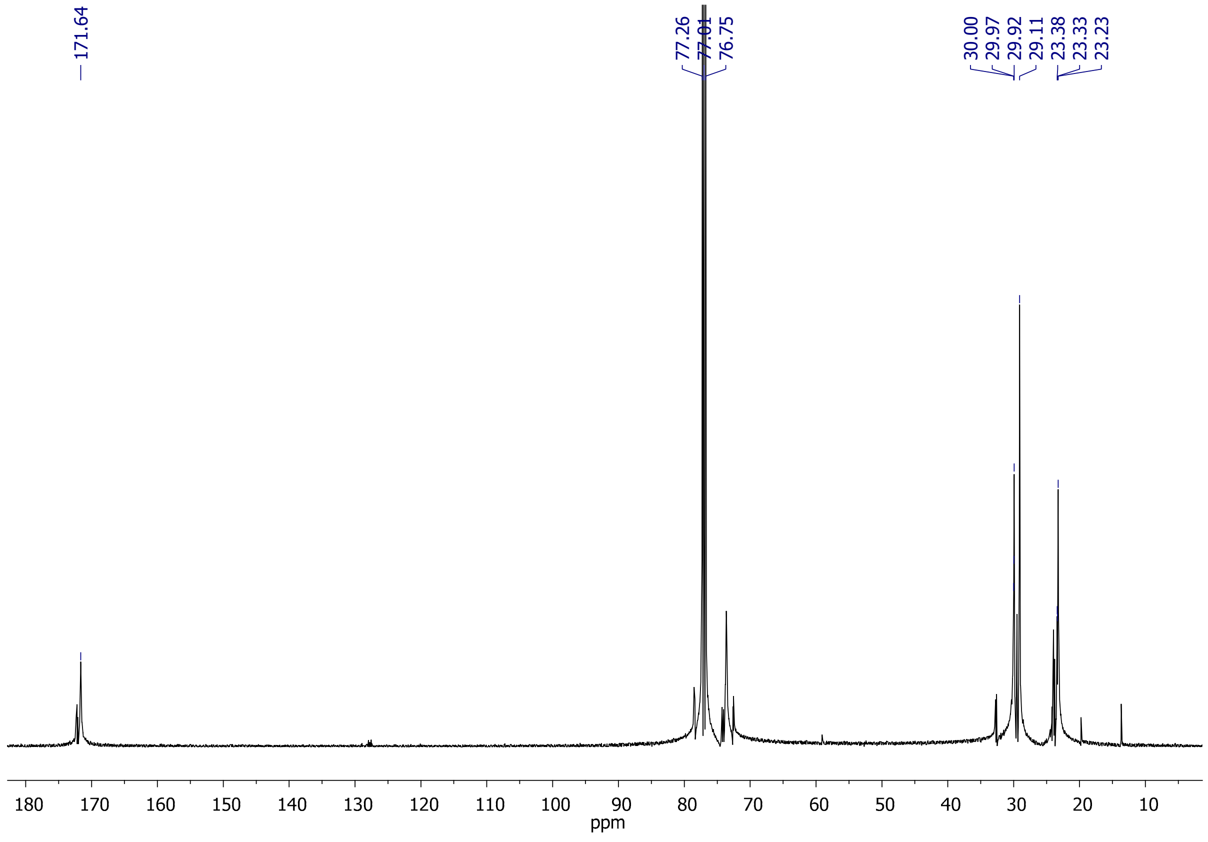

**Figure S6.** IR spectra of: a) poly(SA-*alt*-CHO) (**11**), b) SA (**8**)

**a)**

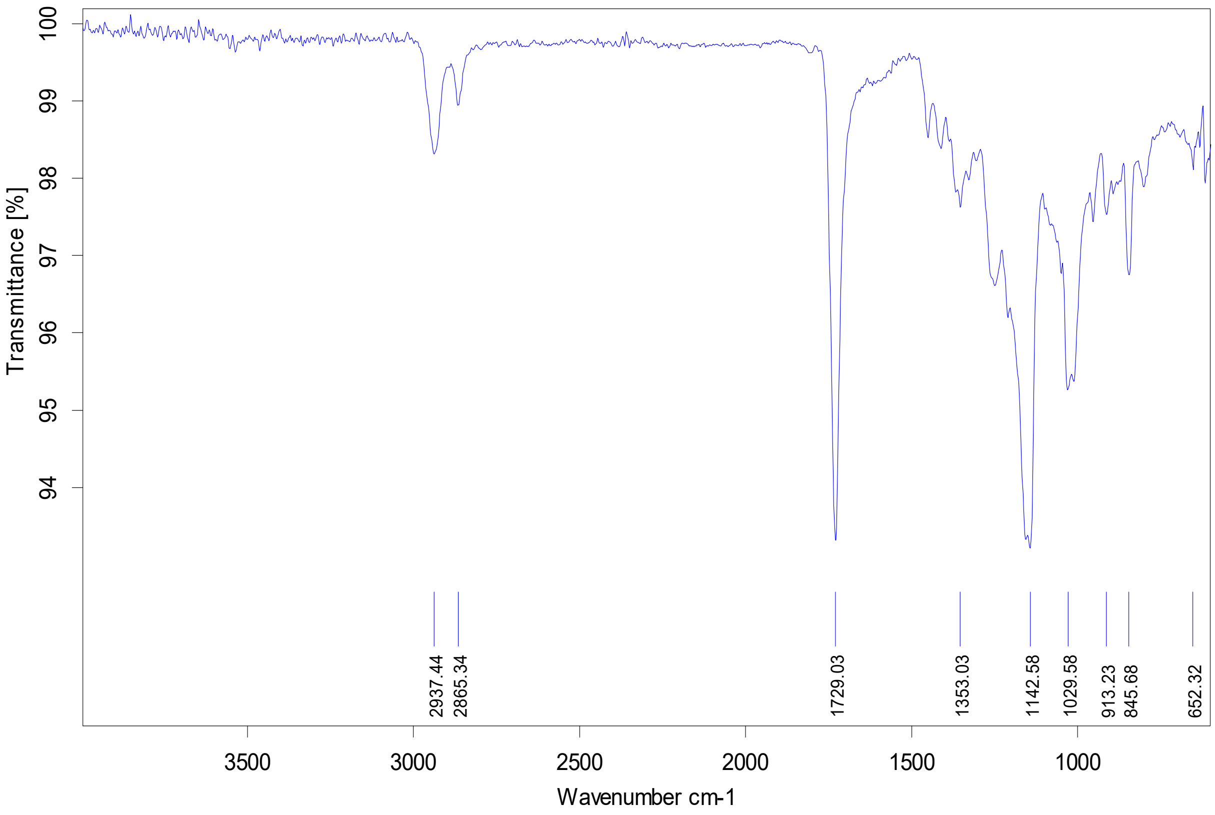


**b)**

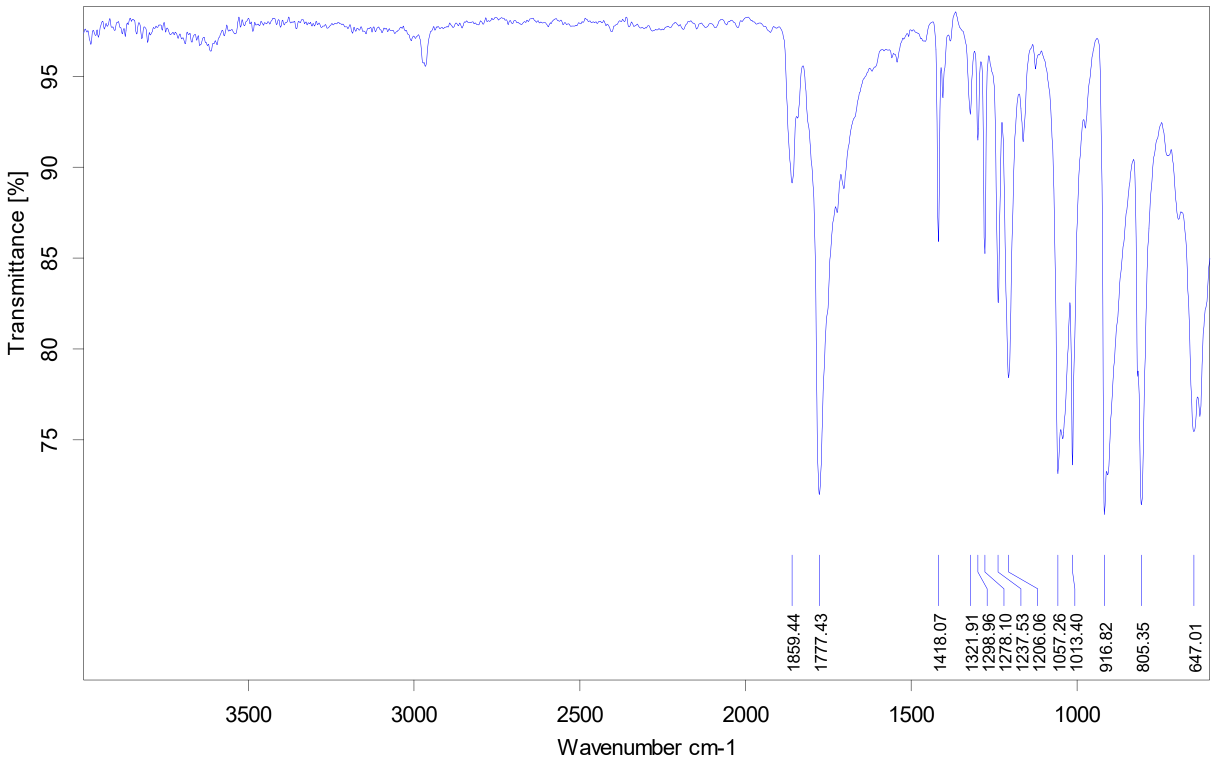

**Figure S7.** $^{1}$H NMR spectrum of poly(MA-*alt*-CHO) (**12**)

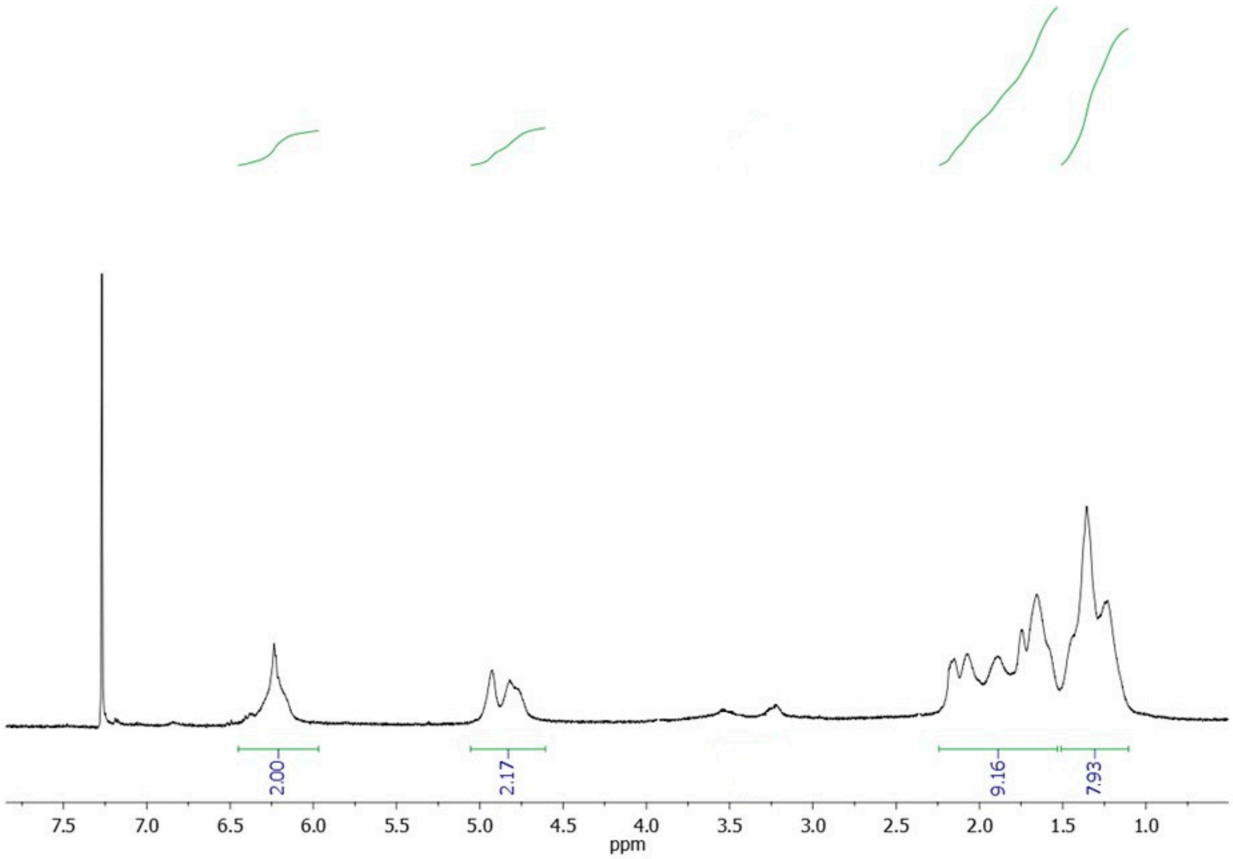


**Figure S8**. $^{13}$C{$^{1}$H} NMR spectrum of poly(MA-*alt*-CHO) (**12**)

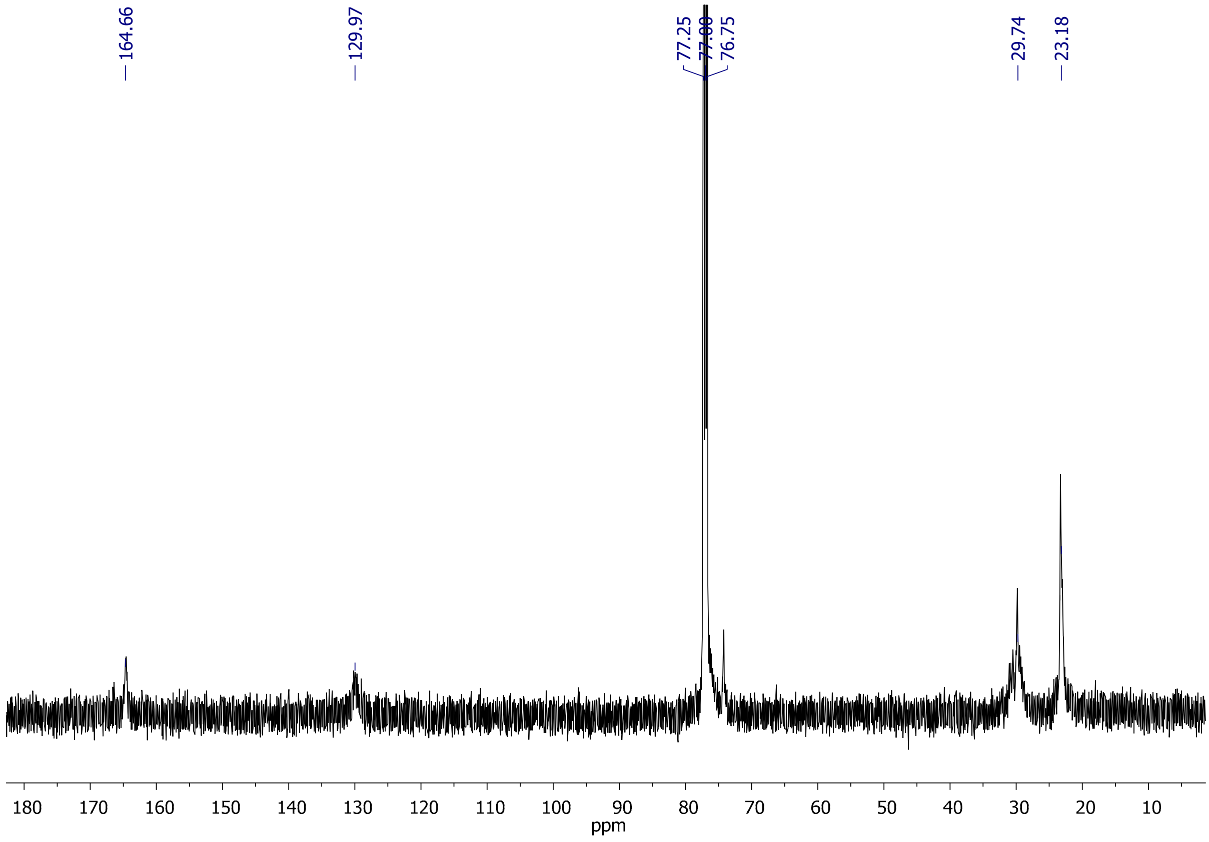

**Figure S9.** IR spectra of: a) poly(MA-*alt*-CHO) (**12**), b) MA (**9**)

**a)**

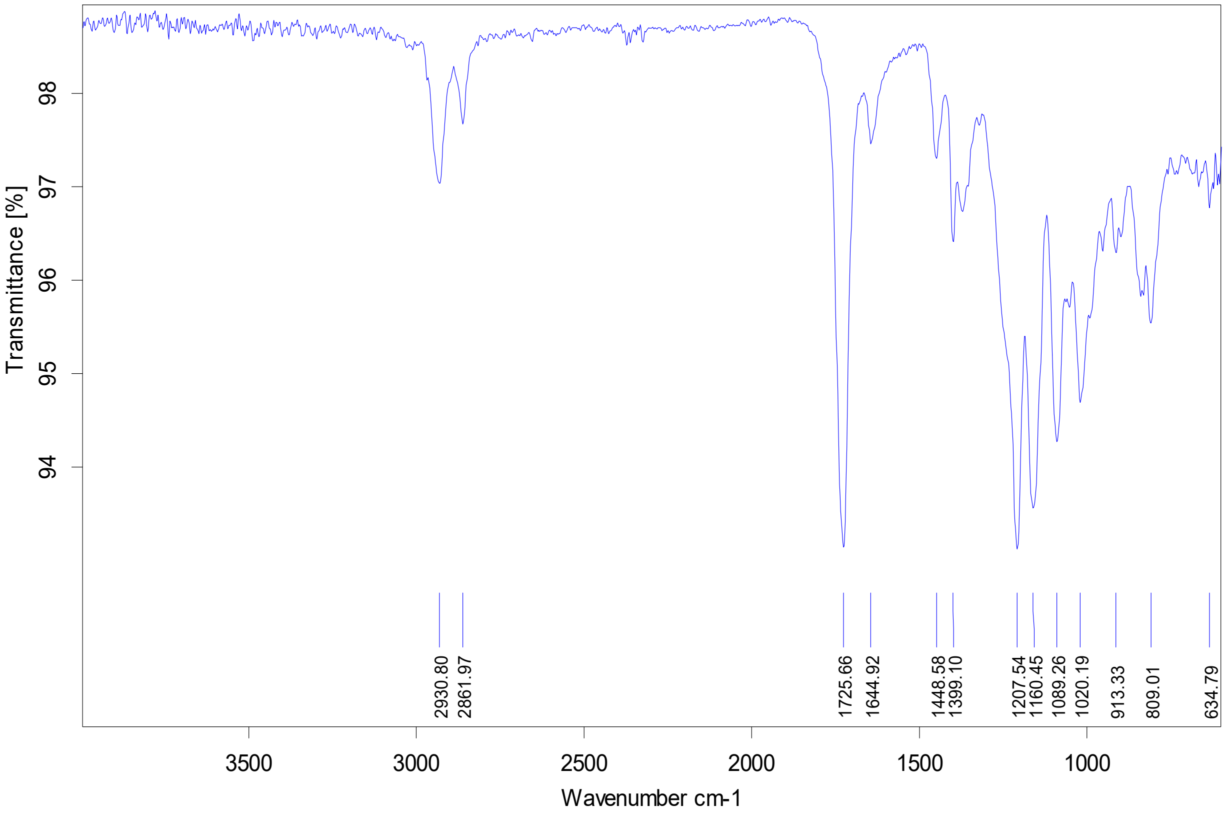


**b)**

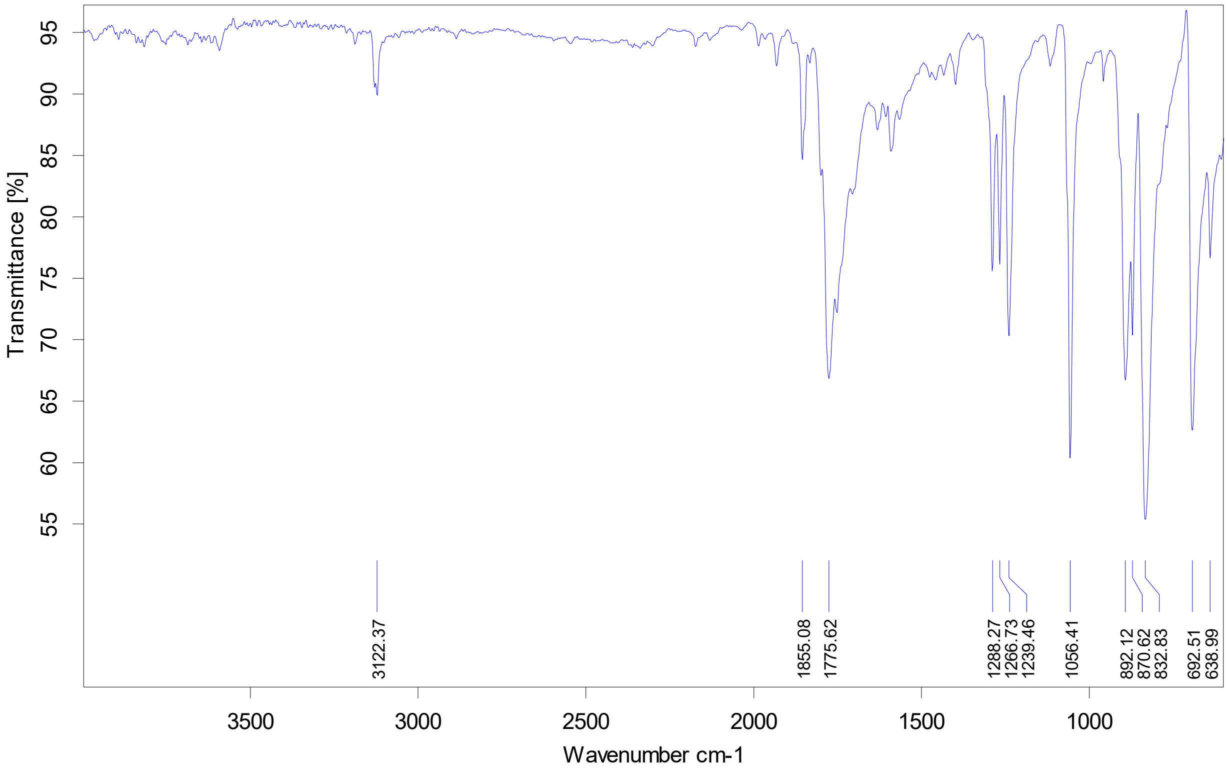

**Figure S10.** $^{1}$H NMR spectrum of poly(NA-*alt*-CHO) (**13**)

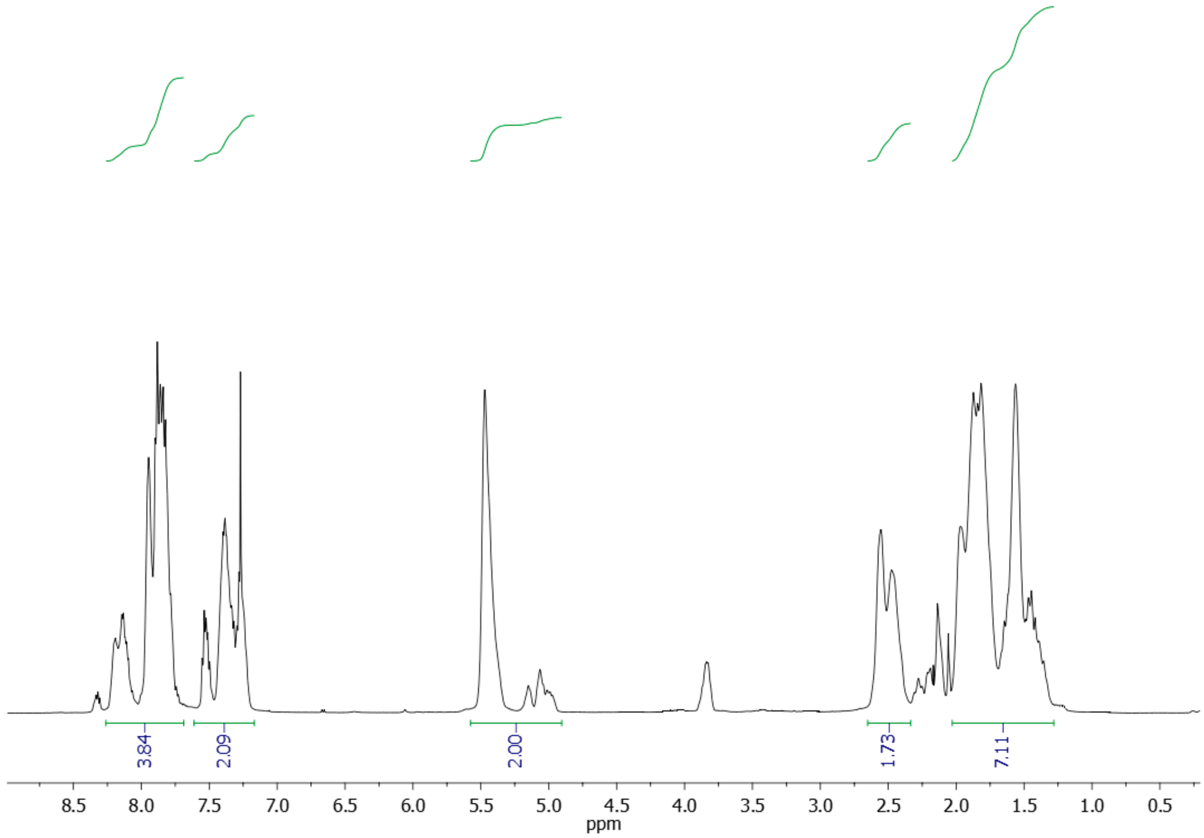


**Figure S11**. $^{13}$C{$^{1}$H} NMR spectrum of poly(NA-*alt*-CHO) (**13**)

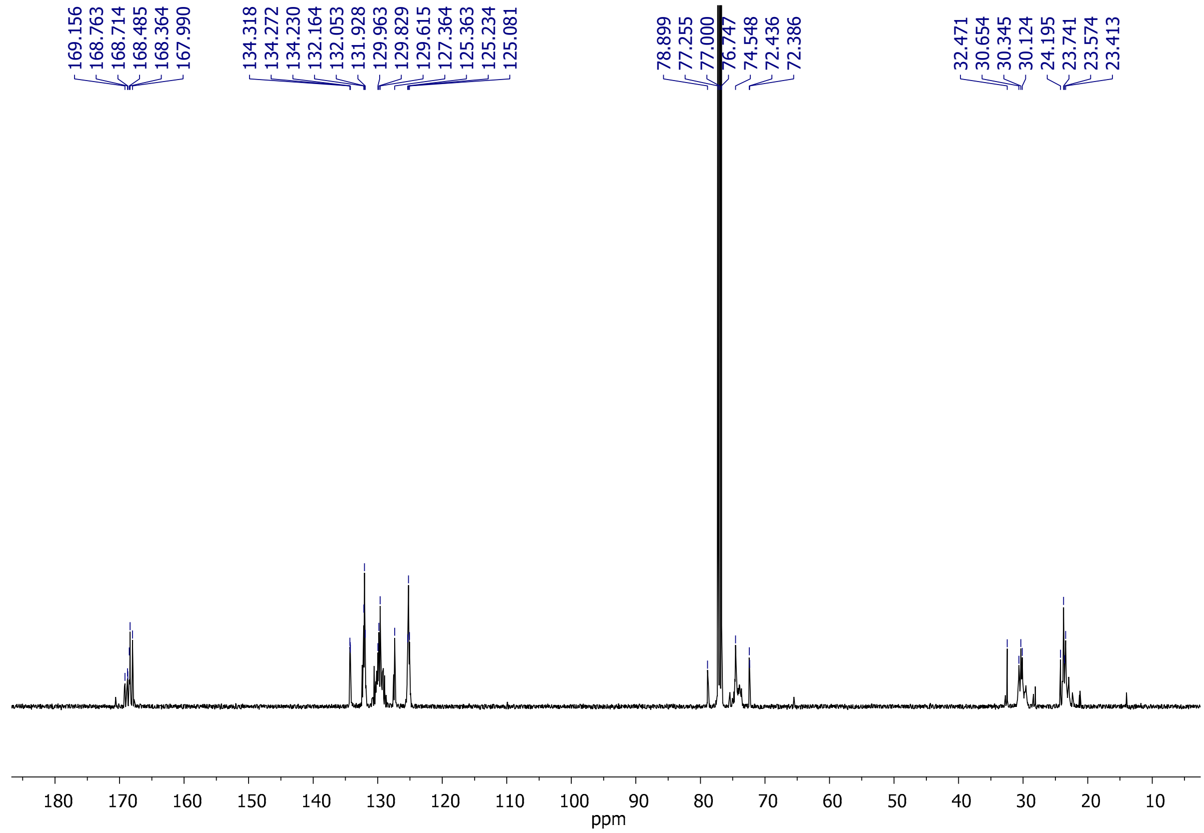

**Figure S12.** IR spectra of: a) poly(NA-*alt*-CHO) (**13**), b) NA (**10**)

**a)**

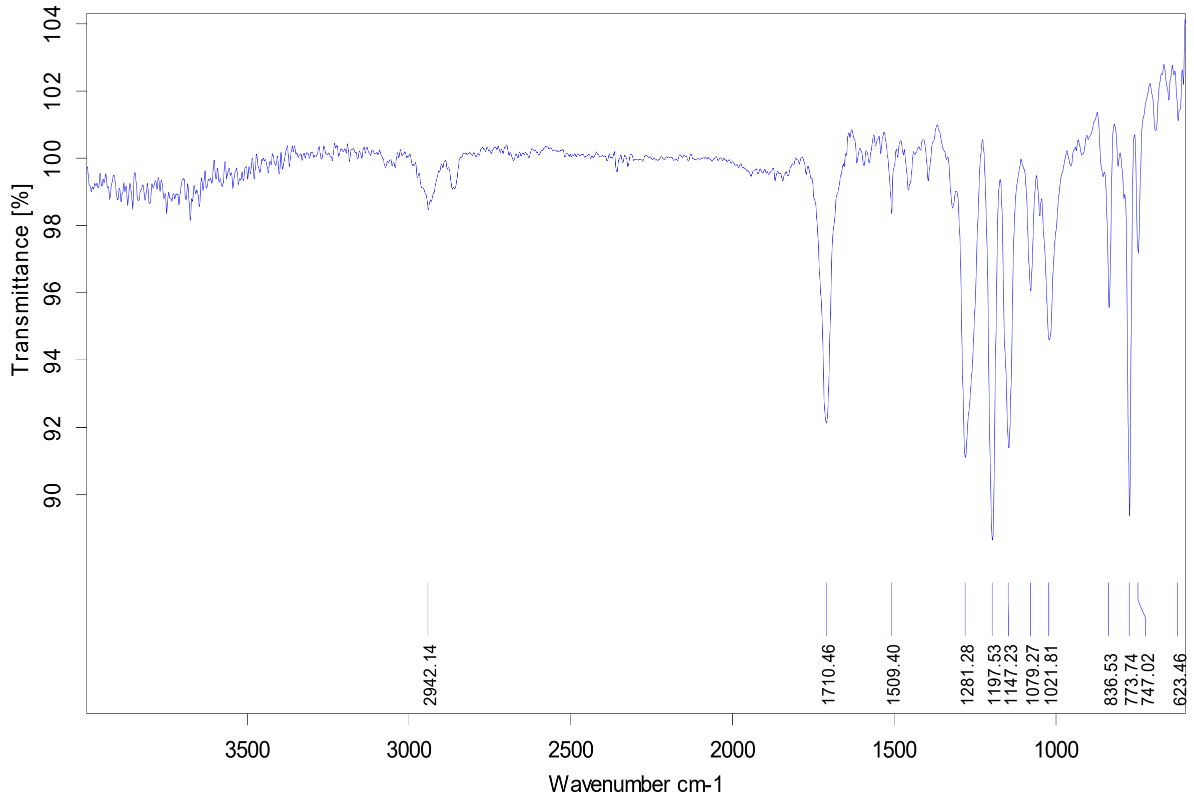


**b)**

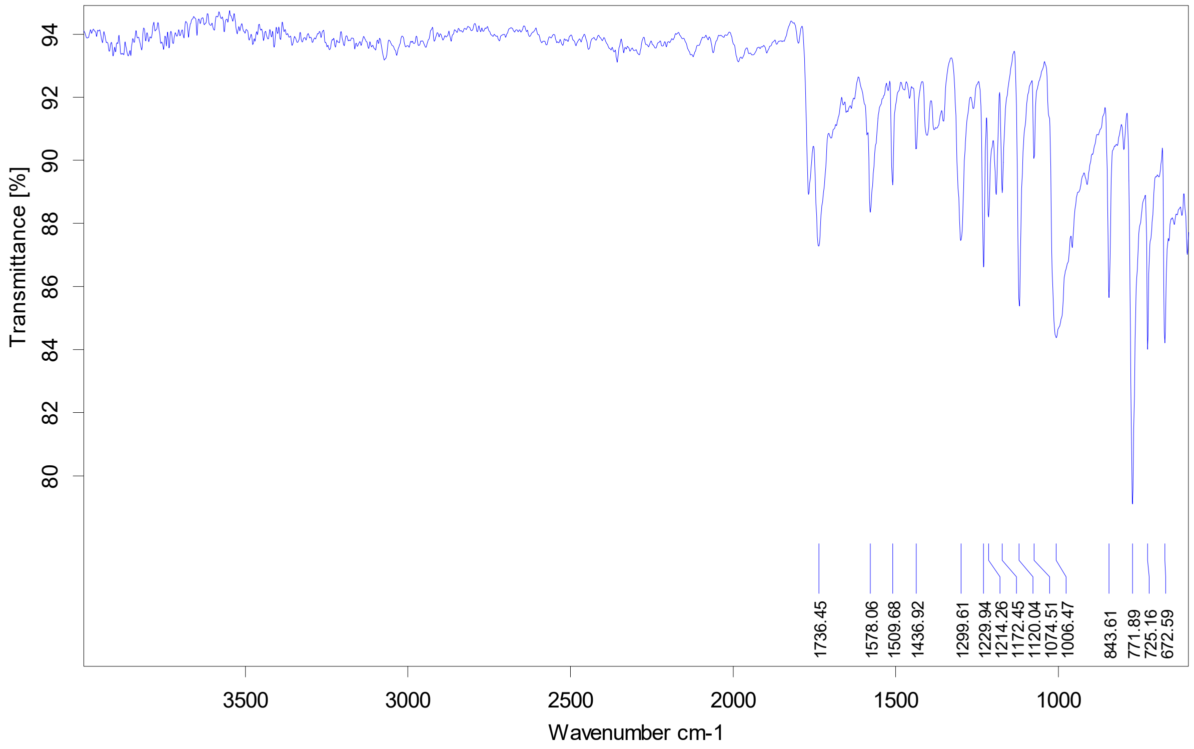

**Figure S13**. GPC profile of poly(PA-*alt*-CHO) (**6**)

## ==== Shimadzu LCsolution Analysis Report ====

C:\LabSolutions\Data\gpcresultados\2019\Felipe\C148.lcd

| | |
|---|---|
| Acquired by | : Admin |
| Sample Name | : C148 |
| Sample ID | : C148 |
| Vail # | : |
| Injection Volume | : 20 uL |
| Data File Name | : C148.lcd |
| Method File Name | : metodo_2017_Marc.lcm |
| Batch File Name | : SingleRun120191016180911.lcb |
| Report File Name | : Default.lcr |
| Data Acquired | : 16/10/2019 17:09:39 |
| Data Processed | : 16/10/2019 17:21:41 |

C:\LabSolutions\Data\gpcresultados\2019\Felipe\C148.lcd

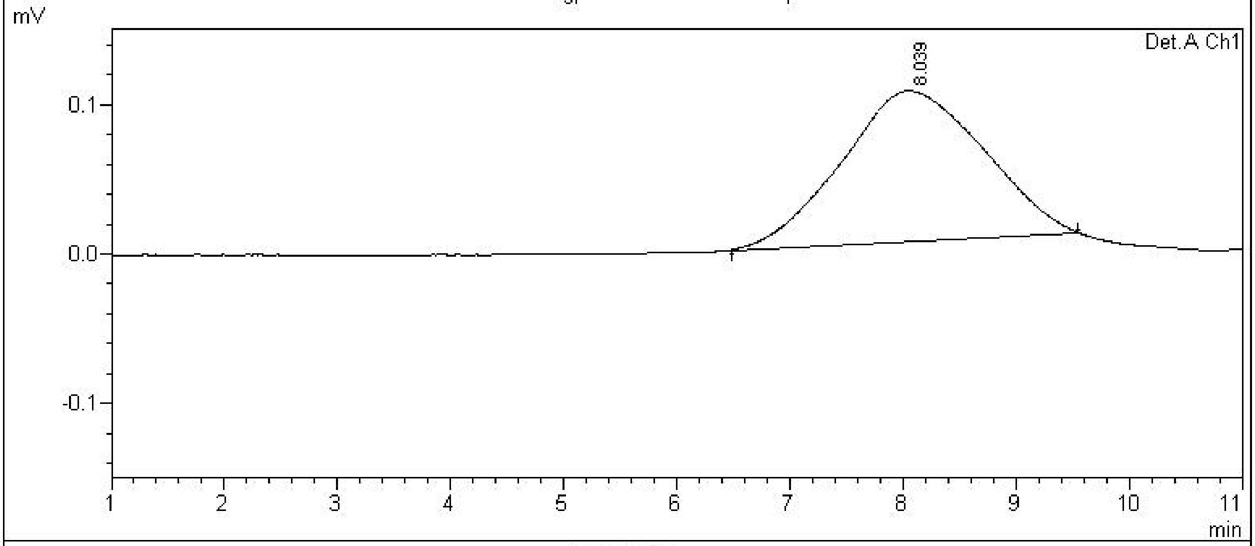


GPC Results

Peak#:1 (Detector A Ch1)

[Average Molecular Weight]

| | |
|---|---|
| Number Average Molecular Weight(Mn) | 2463 |
| Weight Average Molecular Weight(Mw) | 3905 |
| Mw/Mn | 1.58550 |

**Figure S14**. MALDI-ToF spectrum of poly(PA-*alt*-CHO) (**6**)

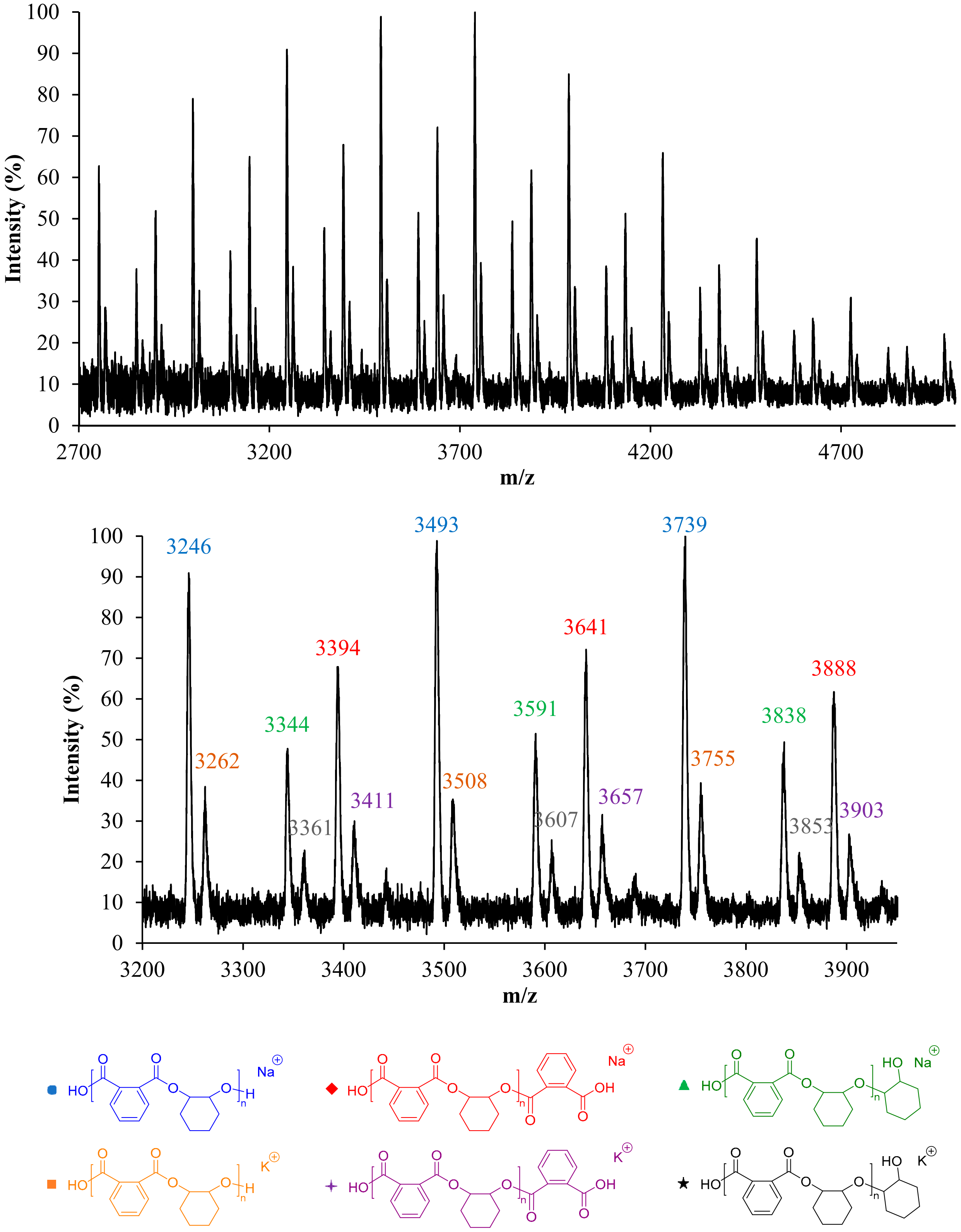

**Figure S15**. TGA analysis of poly(PA-*alt*-CHO) (**6**)

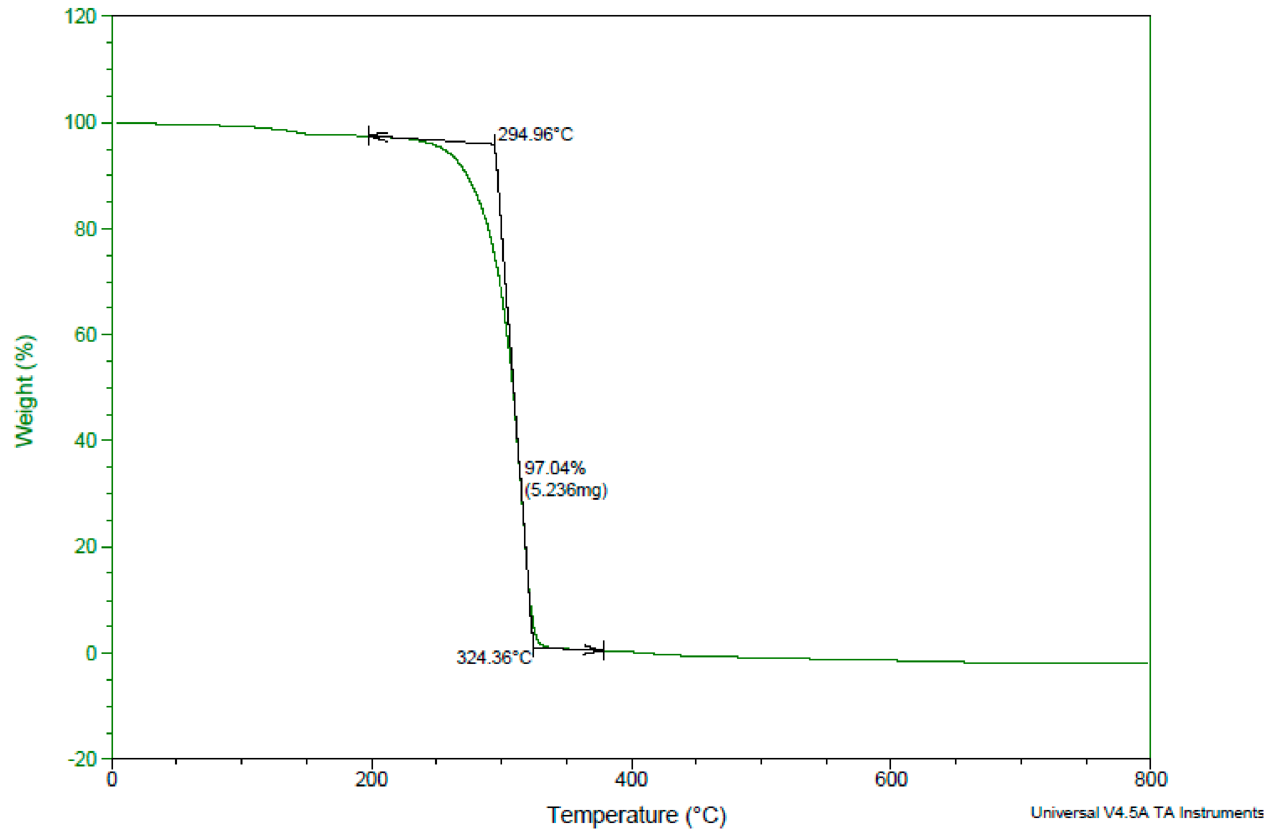


**Figure S16**. DSC analysis of poly(PA-*alt*-CHO) (**6**)

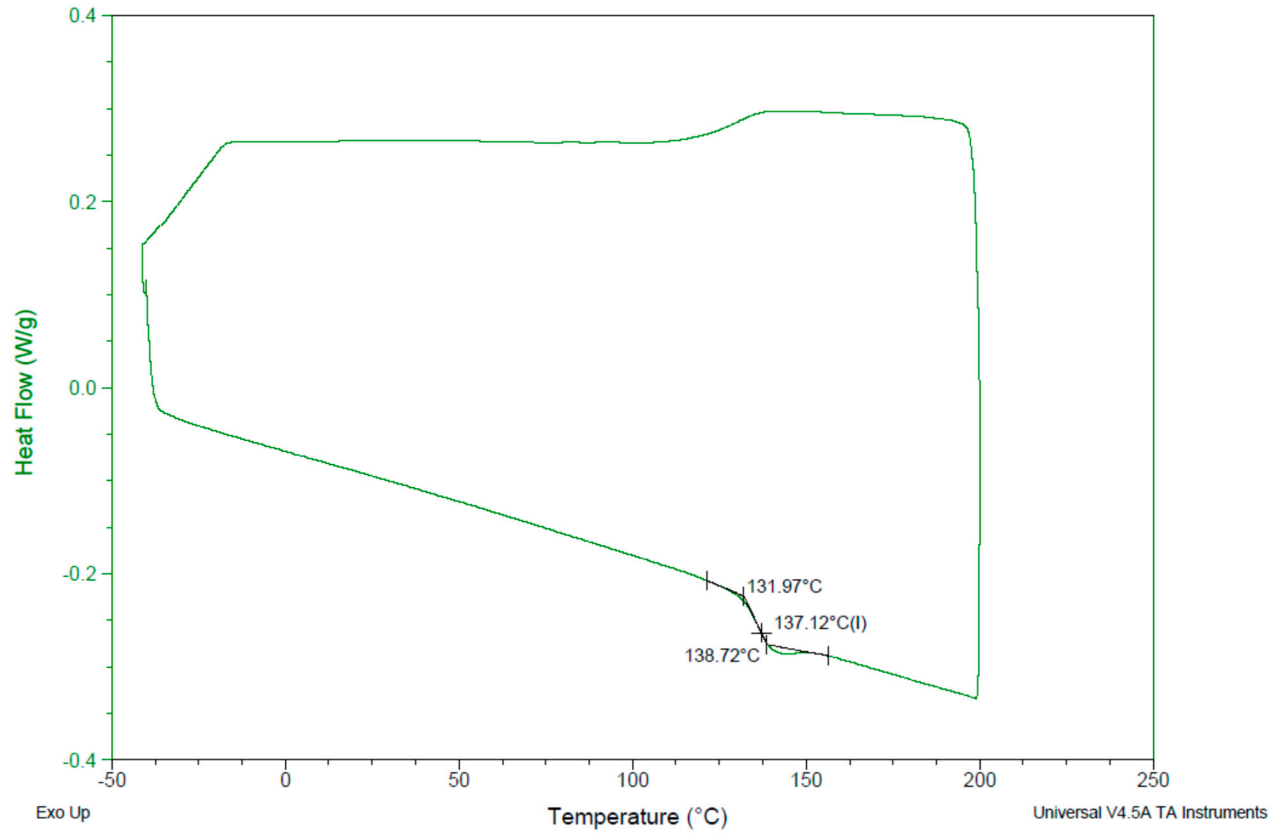

**Figure S17**. TGA analysis of poly(SA-*alt*-CHO) (**11**)

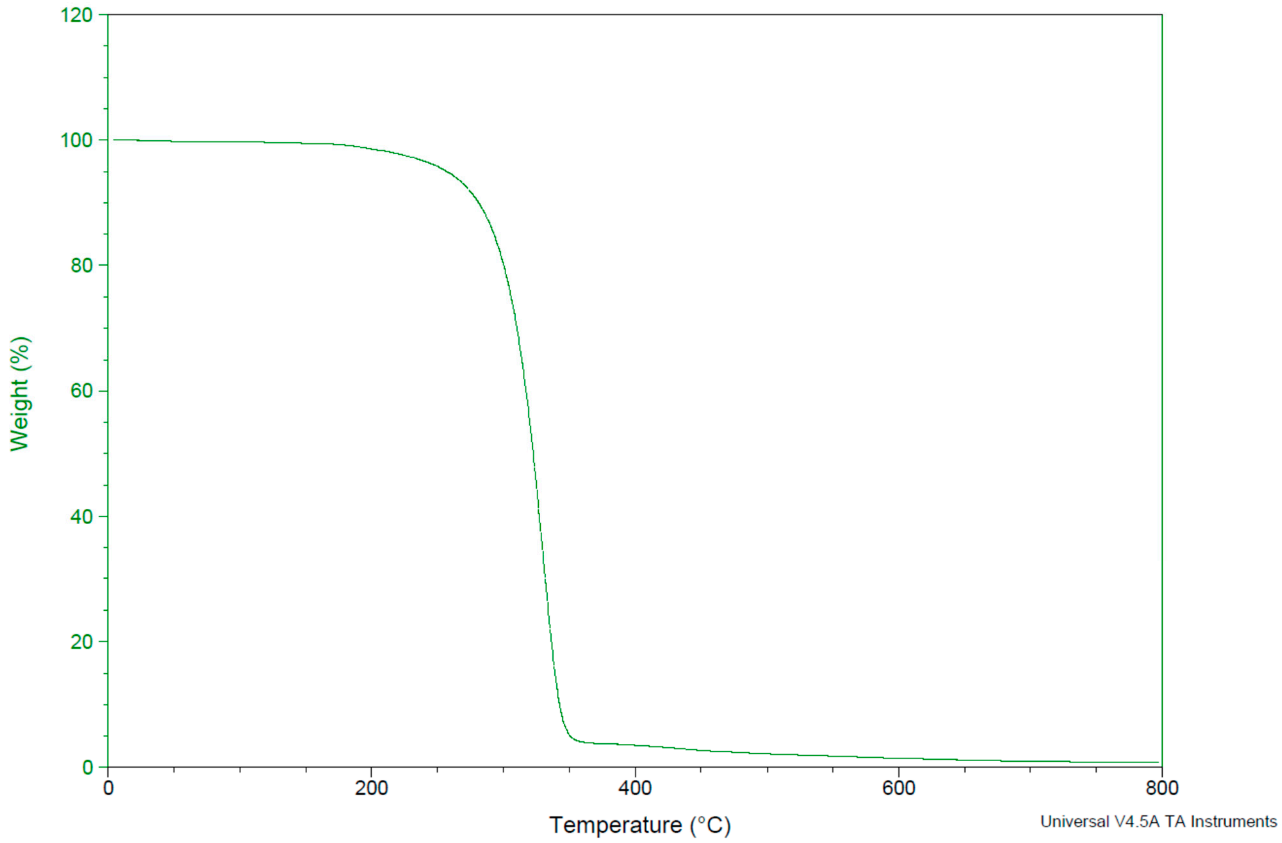


**Figure S18**. DSC analysis of poly(SA-*alt*-CHO) (**11**)

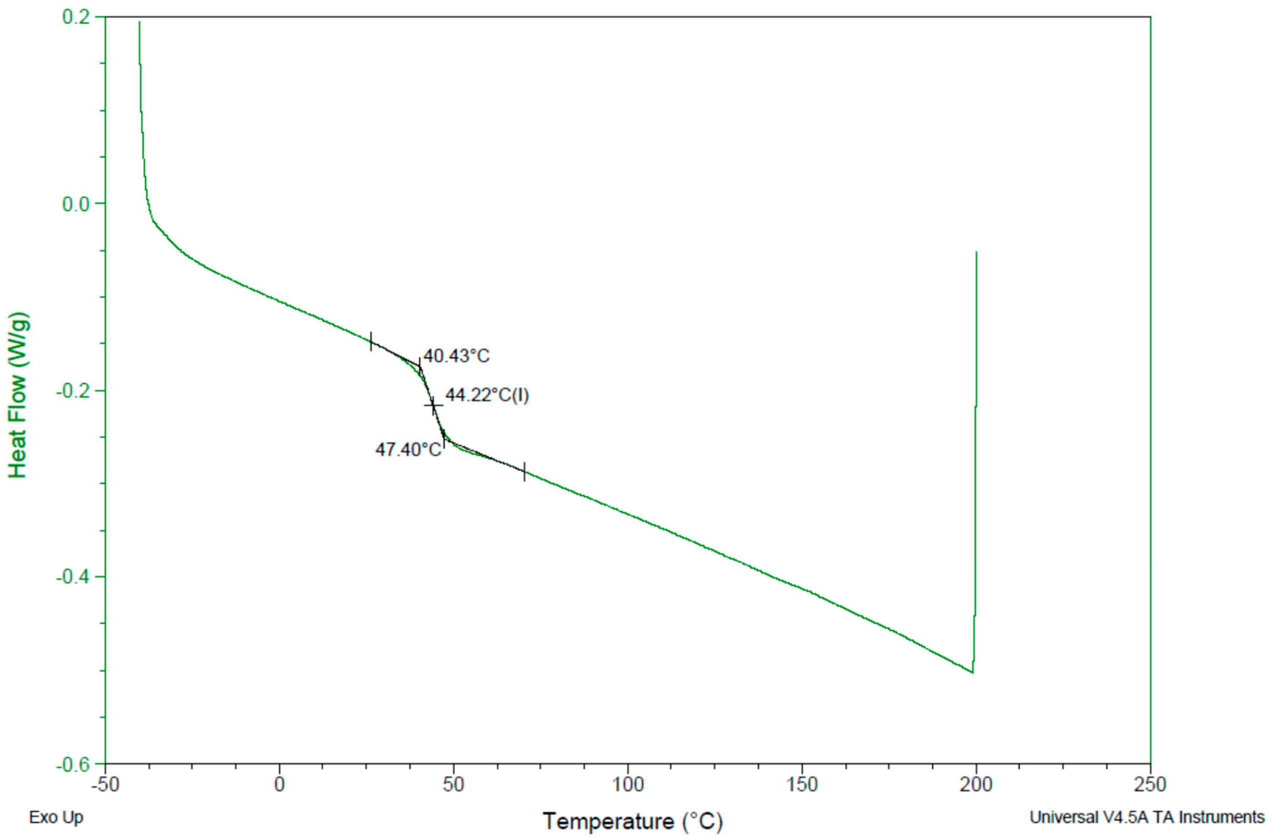

**Figure S19**. TGA analysis of poly(MA-*alt*-CHO) (**12**)

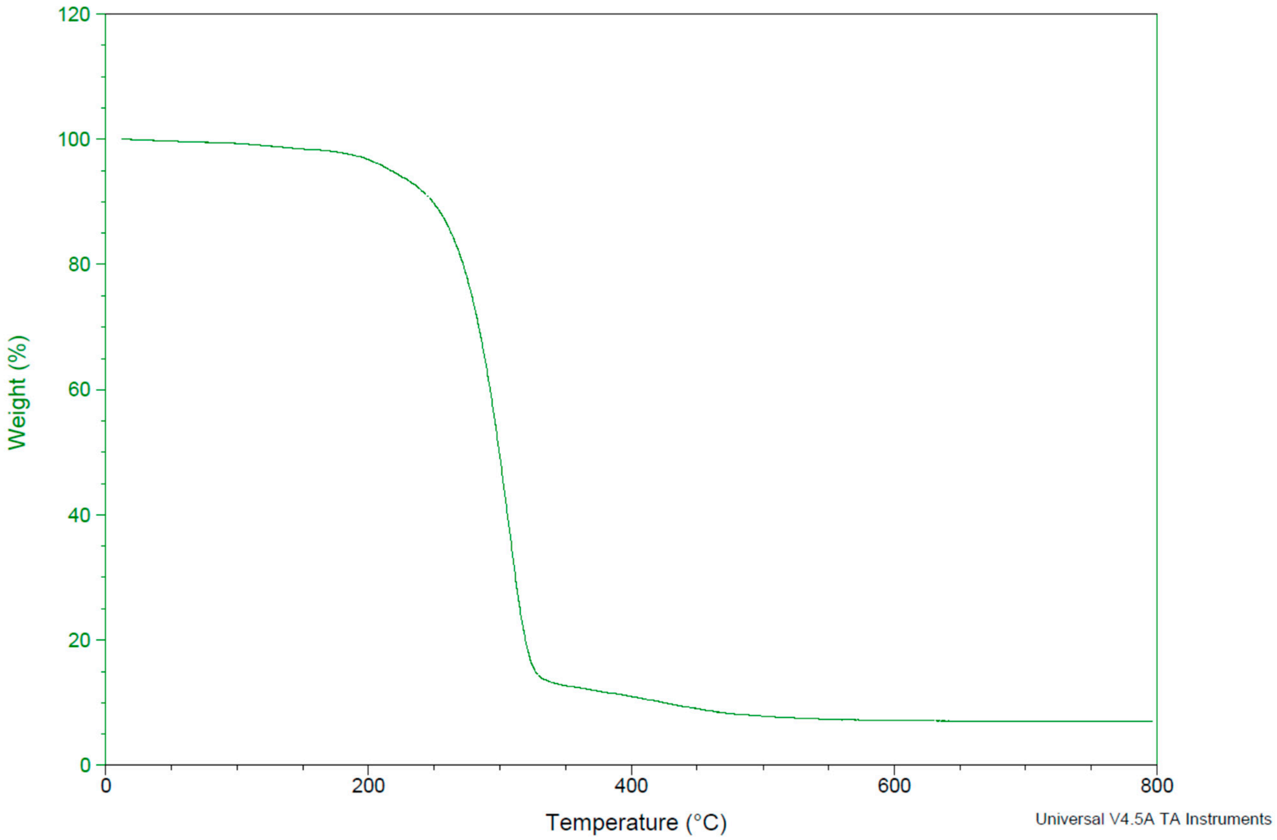


**Figure S20**. DSC analysis of poly(MA-*alt*-CHO) (**12**)

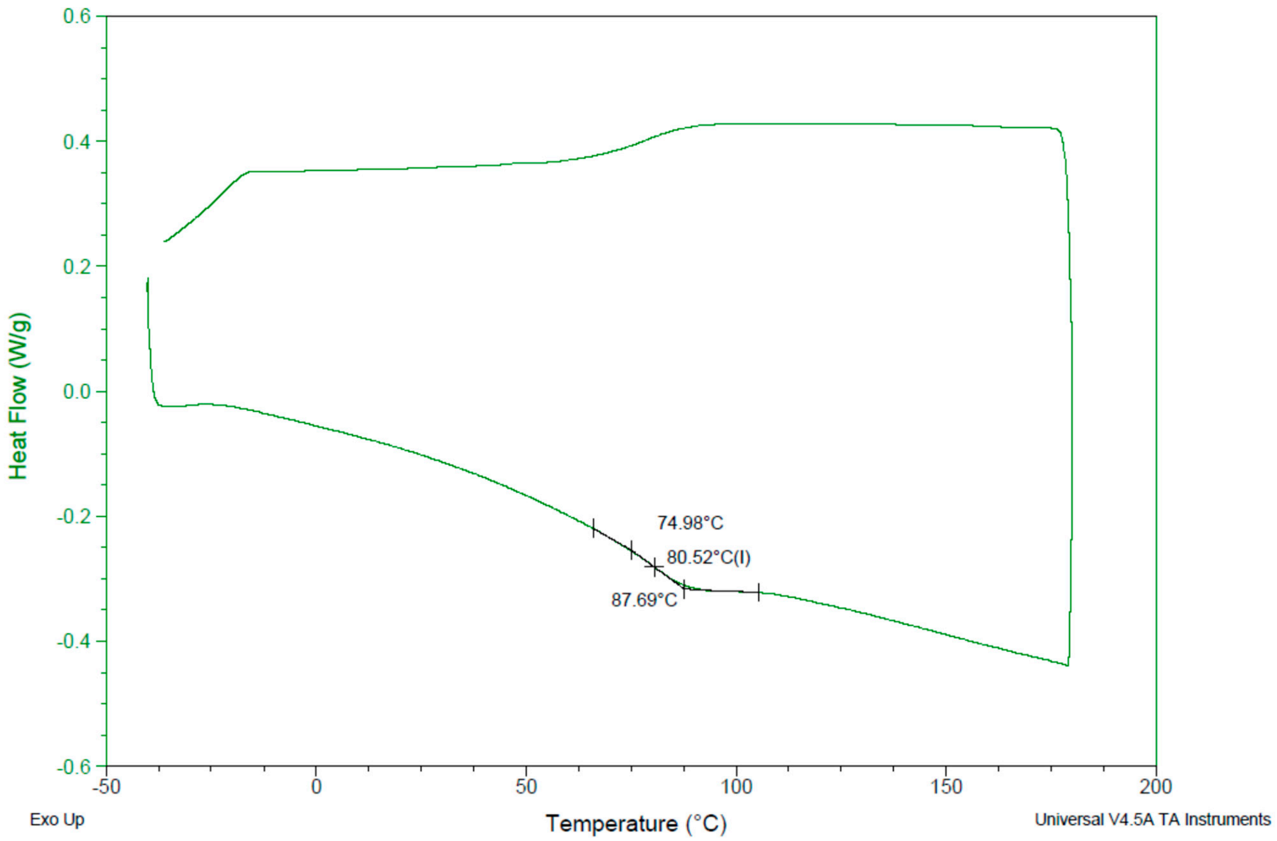

**Figure S21**. TGA analysis of poly(NA-*alt*-CHO) (**13**)

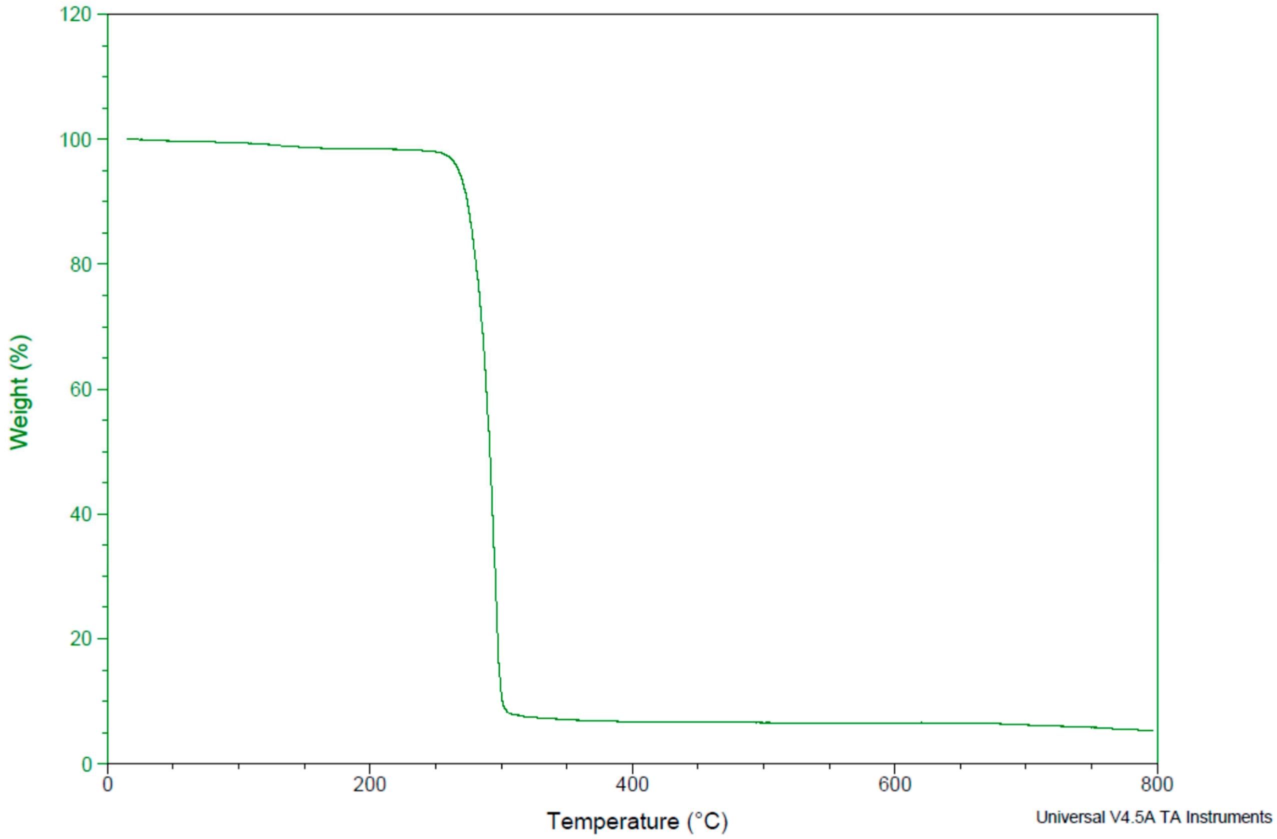


**Figure S22**. DSC analysis of poly(NA-*alt*-CHO) (**13**)

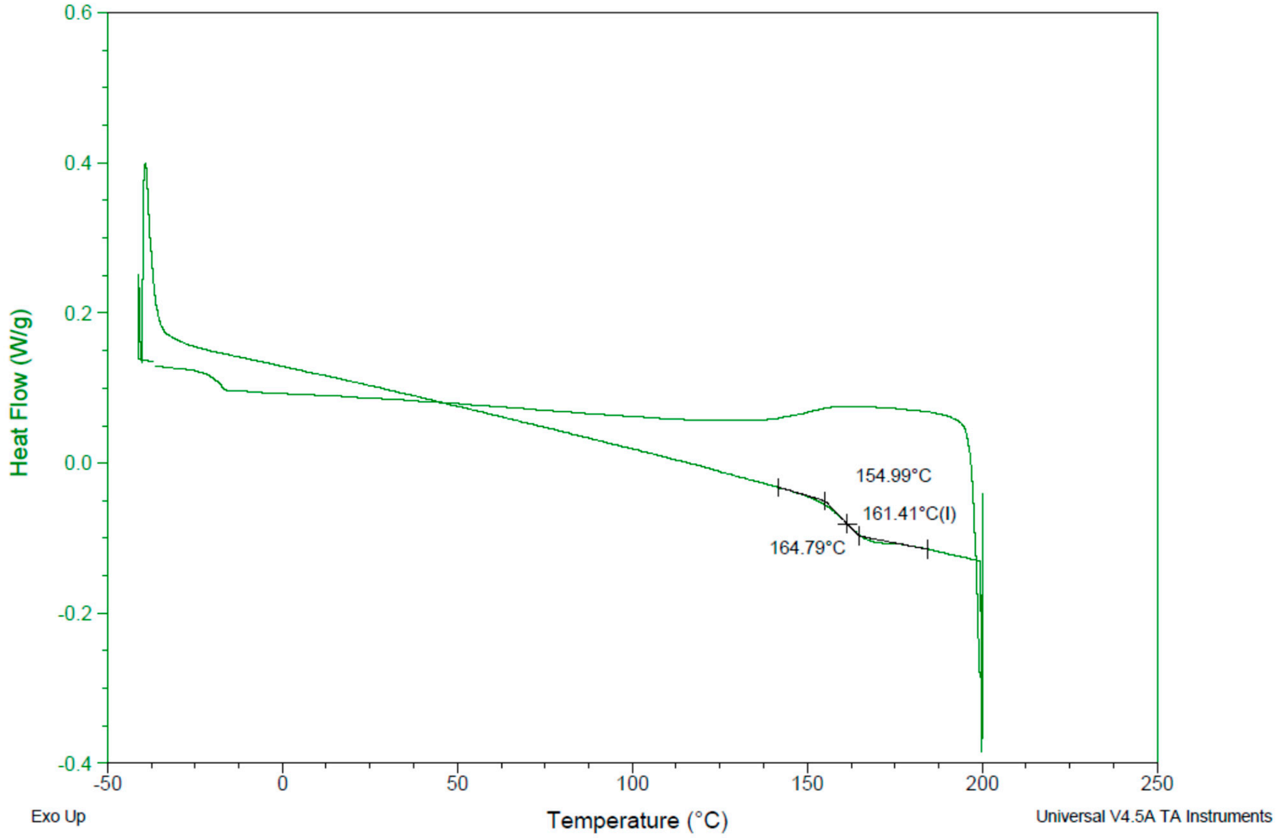

Supplement: Supplementary file 1 [file polymers-13-01651-s001.zip › polymers-1227190-supplementary.pdf]
